# Development of new method to enrich human iPSC-derived renal progenitors using cell surface markers

**DOI:** 10.1038/s41598-018-24714-3

**Published:** 2018-04-23

**Authors:** Azusa Hoshina, Tatsuya Kawamoto, Shin-Ichi Sueta, Shin-Ichi Mae, Toshikazu Araoka, Hiromi Tanaka, Yasunori Sato, Yukiko Yamagishi, Kenji Osafune

**Affiliations:** 1 Center for iPS Cell Research and Application (CiRA), Kyoto University, Kyoto, Japan; 2 Drug Discovery Research, Astellas Pharma Inc, Tsukuba, Ibaraki, Japan; 3 Department of Global Clinical Research, Graduate School of Medicine, Chiba University, 1-8-1 Inohana, Chuo-ku, Chiba, Japan

**Keywords:** Induced pluripotent stem cells, Stem-cell differentiation

## Abstract

Cell therapy using renal progenitors differentiated from human embryonic stem cells (hESCs) or induced pluripotent stem cells (hiPSCs) has the potential to significantly reduce the number of patients receiving dialysis therapy. However, the differentiation cultures may contain undifferentiated or undesired cell types that cause unwanted side effects, such as neoplastic formation, when transplanted into a body. Moreover, the hESCs/iPSCs are often genetically modified in order to isolate the derived renal progenitors, hampering clinical applications. To establish an isolation method for renal progenitors induced from hESCs/iPSCs without genetic modifications, we screened antibodies against cell surface markers. We identified the combination of four markers, CD9^−^CD140a^+^CD140b^+^CD271^+^, which could enrich OSR1^+^SIX2^+^ renal progenitors. Furthermore, these isolated cells ameliorated renal injury in an acute kidney injury (AKI) mouse model when used for cell therapy. These cells could contribute to the development of hiPSC-based cell therapy and disease modeling against kidney diseases.

## Introduction

Dialysis therapy and renal transplantation are currently the only effective options to treat stage 5 chronic kidney disease (CKD). Renal transplantation is effective but uncommon because of a lack of donors, and dialysis therapy reduces patient quality of life (QOL). Further, an increasing number of CKD patients is causing growing economic burden to health care systems^1^. In order to solve these problems, regenerative medicine strategies using embryonic renal progenitors differentiated from human embryonic stem cells (hESCs) or induced pluripotent stem cells (hiPSCs) have attracted attention.

Differentiation methods for renal progenitor cells from hESCs/iPSCs have mimicked normal kidney development^2,3^. The mammalian adult kidney metanephros is formed at the posterior end of intermediate mesoderm (IM)^4^. A nuclear transcription factor, Odd-skipped related 1 (Osr1), is one of the earliest markers specific for the IM, whose expression starts early (embryonic day (E) 7.5)^5^. Osr1 knockout mice lack renal structures due to the failure to form the IM^6,7^. A homeodomain transcriptional regulator, Six2, is expressed in cap mesenchyme (CM) derived from metanephric mesenchyme (MM). Cells expressing Six2 represent a multipotent, self-renewing progenitor population that can generate all cell types of the main body of the nephron^8,9^. Inactivation of Six2 results in premature and ectopic renal vesicles, leading to a reduced number of nephron and to renal hypoplasia^10^. Overall, in contemporary models, Osr1 and Six2 are required to maintain renal progenitor cells during kidney organogenesis^11^.

Several groups have reported renal progenitor induction from hESCs/iPSCs^12–15^. Taguchi *et al*. demonstrated the induction of SIX2^+^ renal progenitor cells from hiPSCs by aggregate cultures, and these renal progenitor cells exhibited tubulogenesis and podocyte formation when cocultured with mouse embryonic spinal cords^12^. Takasato *et al*. produced nephron structures from hiPSCs by organoid cultures and induced SIX2^+^ renal progenitor cells during the induction^13^. Morizane *et al*. reported the induction of SIX2^+^ renal progenitor cells from hiPSCs by 2D cultures, and that the cells went on to form nephron structures after being transferred to 3D culture conditions^14^. They also showed an elevation of *OSR1* mRNA expression in the induced SIX2^+^ renal progenitor cells by qRT-PCR.

Despite the above success, the induced cells are not suitable for clinical applications, because the induction rates of SIX2^+^ renal progenitors suggested that other lineage cells as well as undifferentiated cells might be mixed in the differentiation cultures. These contaminating cells could cause neoplastic formations and other unexpected side effects. Previously, we reported a protocol for differentiating hiPSCs into OSR1^+^SIX2^+^ renal progenitors^15^. Although the induction rate was low at around 40%, the progenitor cells showed therapeutic effects by transplantation into the renal subcapsule of acute kidney injury (AKI) model mice. However, because both progenitor markers are nuclear transcriptional factors, the hiPSCs were genetically modified to express OSR1-green fluorescent protein (GFP) and SIX2-tdTomato for isolation of the cells, meaning the cells cannot be used for clinical applications.

Here, we developed an isolation method for renal progenitors by flow cytometry that avoids genome editing and uses monoclonal antibodies against cell surface markers. We screened monoclonal antibodies against cell surface markers that isolate OSR1^+^SIX2^+^ renal progenitors by flow cytometry and identified three positive and three negative selection markers. We then identified the combination of CD9^−^CD140a^+^CD140b^+^CD271^+^ as surface markers for renal progenitors derived from hiPSCs that have therapeutic potential for AKI in mice. The isolation method established in this study can provide a tool for efficient and safe cell therapy and disease modeling.

## Results

### Screening selectable markers to concentrate OSR1^+^SIX2^+^ cells differentiated from hiPSCs

**Figure 1:**
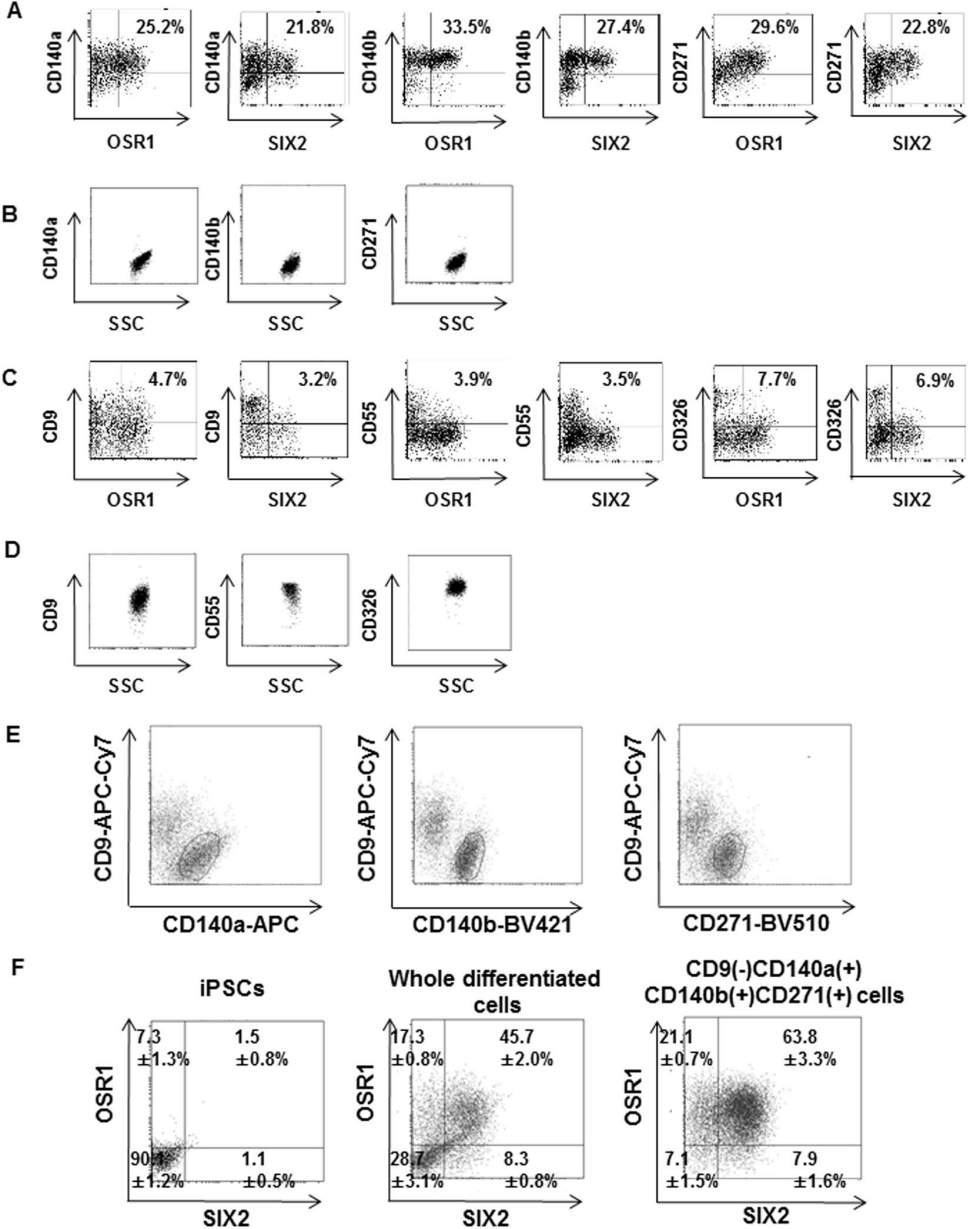
Flow cytometric analysis and characterization of surface markers that can concentrate OSR1^+^SIX2^+^ cells from differentiation culture. (**A**) Positive selectable markers that detect OSR1^+^ and SIX2^+^ cells. (**B**) These positive selectable markers do not detect undifferentiated hiPSCs. (**C**) Negative selectable markers that are negatively correlated with OSR1^+^ or SIX2^+^ cells. (**D**) These negative selectable markers are expressed in undifferentiated hiPSCs. (**E**) Differentiated cells fractioned with antibodies directed against CD9, CD140a, CD140b and CD271. (**F**) Flow cytometric analysis of undifferentiated hiPSCs (left), whole differentiated cells before isolation (center) and isolated cells fractioned with gates of CD9^−^CD140a^+^, CD9^−^CD140b^+^ and CD9^−^CD271^+^ (right) for OSR1 and SIX2. Results of the antibody screening are shown in (**A**) and (**C**). Representative data from at least three independent experiments are shown in (**B**), (**D**) and (**E**). The data from three independent experiments are presented as the mean ± SE (n = 3) in (**F**).

The screening of monoclonal antibodies against cell surface markers was performed on the differentiated cells around day 28 of our differentiation protocol^15^ using commercially available screening panels that included 242 antibodies and flow cytometry. To search selectable surface markers for OSR1^+^SIX2^+^ cells in whole differentiated cells without purification, we used an OSR1-GFP/SIX2-tdTomato double knock-in hiPSC line we had previously established from a fibroblast-derived hiPSC line, 201B7^15^. First, we picked up three cell surface markers (CD140a, CD140b and CD271) that could detect OSR1^+^ and SIX2^+^ cells (Fig. 1A), but not undifferentiated hiPSCs (Fig. 1B). We next picked up an additional three cell surface markers (CD9, CD55 and CD326) that were negatively correlated with OSR1^+^ and SIX2^+^ cells (Fig. 1C) and expressed in hiPSCs (Fig. 1D), enabling us to exclude undifferentiated cells from the differentiated cultures.

To efficiently concentrate OSR1^+^SIX2^+^ cells, we tested various combinations of these selectable markers (Table S1). As a result, we chose the combination of CD9, CD140a, CD140b and CD271 as the most efficient to obtain OSR1^+^SIX2^+^ cells (Figs 1E and S1). Fractionated cells by CD9^−^CD140a^+^CD140b^+^CD271^+^ were isolated and analyzed to confirm the enrichment of OSR1^+^SIX2^+^ cells with these markers by flow cytometry. The percentage of CD9^−^CD140a^+^CD140b^+^CD271^+^ cells in each fraction was as follows: OSR1^−^SIX2^−^ fraction, 7.1 ± 1.5% (n = 3); OSR1^+^SIX2^−^ fraction, 21.1 ± 0.7% (n = 3); OSR1^−^SIX2^+^ fraction, 7.9 ± 1.6% (n = 3); and OSR1^+^SIX2^+^ fraction, 63.8 ± 3.3% (n = 3). Thus, OSR1^+^SIX2^+^ cells compared with whole differentiated cells were markedly enriched in CD9^−^CD140a^+^CD140b^+^CD271^+^ cells (Fig. 1F).

We next examined these cell surface markers on cells differentiated from another hiPSC line, 585A1, which was derived from the peripheral blood mononuclear cells of a healthy Japanese donor^16^. Because CD140a^+^, CD140b^+^ and CD271^+^ cells were induced at low efficiency (7.6 ± 1.4%, 11.2 ± 1.9% and 10.7 ± 2.4%, respectively (n = 3)), the induction efficiency of CD9^−^CD140a^+^CD140b^+^CD271^+^ cells was low at 5.0 ± 1.2% (n = 3) (Fig. S2). Nevertheless, we could obtain CD9^−^CD140a^+^CD140b^+^CD271^+^ renal progenitors from 585A1 cells.

### Isolation of hiPSC-derived renal progenitors using cell surface markers

**Figure 2:**
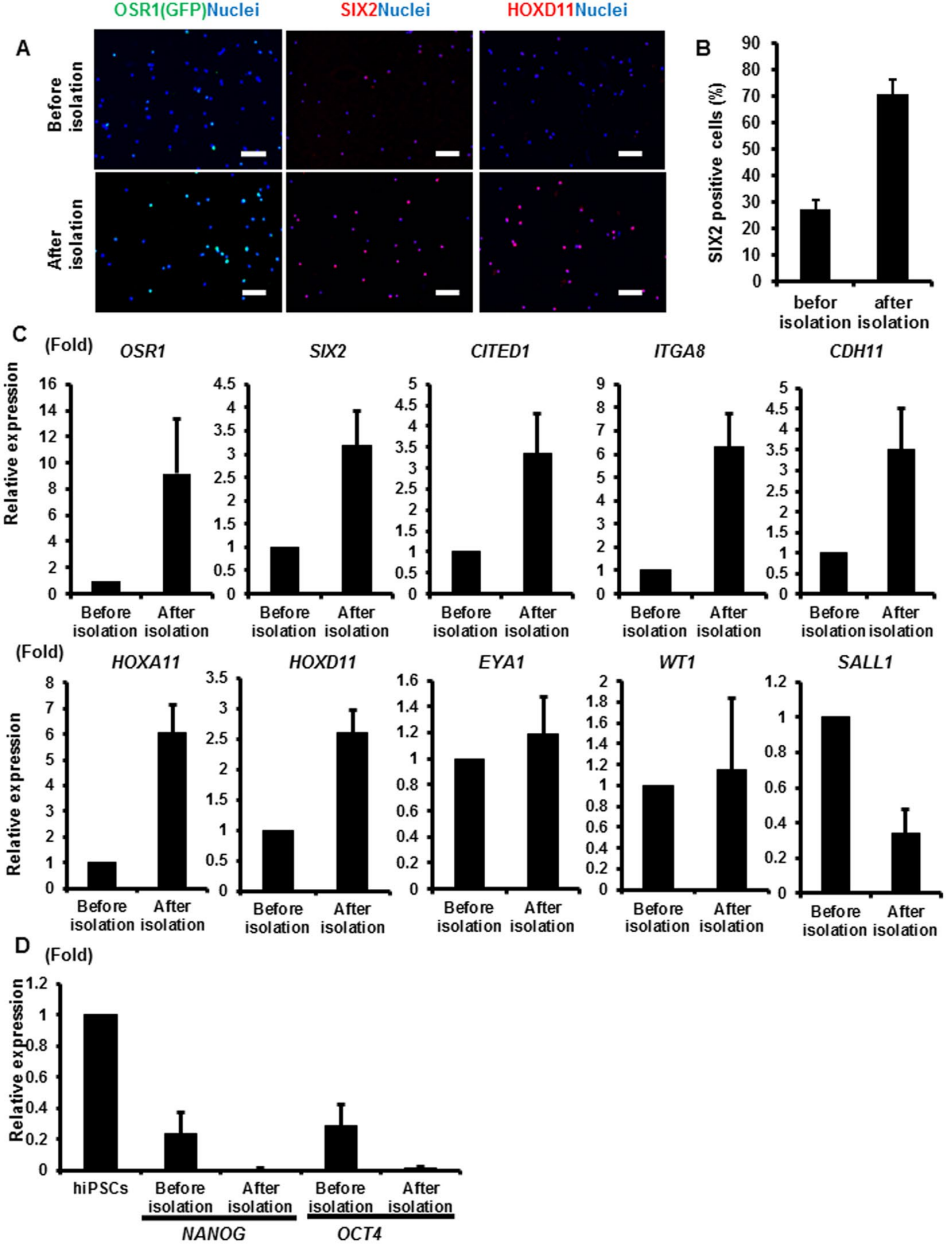
The expression of renal lineage markers in CD9^−^CD140a^+^CD140b^+^CD271^+^ cells isolated from hiPSC differentiation culture. (**A**) Anti-GFP (OSR1), SIX2 and HOXD11 immunostaining images of the cells before isolation (upper panels) and isolated CD9^−^CD140a^+^CD140b^+^CD271^+^ cells (lower panels) on culture days 28 (SIX2) and 30 (OSR1 and HOXD11). Representative images from three independent experiments are shown. Scale bars, 100 µm. (**B**) The induction rates of SIX2^+^ cells in day 28 cells before and after isolation. The data from three randomly chosen fields are presented as the mean ± SE (n = 3). (**C,D**) qRT-PCR analyses of gene expressions in day 28 cells before and after isolation for markers of nephron progenitors (**C**) and undifferentiated cells (**D**). The data from three independent experiments are presented as the mean ± SE (n = 3). *GAPDH* was used as an internal control, and the relative expression levels were normalized to those of day 28 cells before isolation (**C**) and hiPSCs (**D**). ITGA8, integrin alpha 8; CDH11, cadherin 11.

In order to determine whether the hiPSC-derived CD9^−^CD140a^+^CD140b^+^CD271^+^ cells show characteristics of embryonic renal progenitors, the cells were isolated with a flow cytometer. We observed that the ratio of OSR1 (GFP)^+^, SIX2^+^ and HOXD11^+^ cells after isolation was substantially increased compared with that before isolation by immunostaining (Fig. 2A,B). We then analyzed the gene expression levels of *OSR1* and *SIX2* and found an enrichment of CD9^−^CD140a^+^CD140b^+^CD271^+^ cells compared with unsorted cells (Fig. 2C). We also found enrichment in the expression levels of other renal progenitor markers such as *CITED1, ITGA8, CDH11, HOXA11* and *HOXD11* compared with unsorted cells. In contrast, we did not observe a significant increase in the expression levels of *EYA1, WT1* or *SALL1*. Because these genes are expressed during the IM and early MM stages^4^, we assumed that OSR1^+^SIX2^−^ cells included in the unsorted cells might express these markers and explain the absence of enrichment after isolation. As expected, the expression levels of undifferentiated markers, such as *NANOG* and *OCT4*, were lower after isolation (Fig. 2D). We also confirmed that the isolated cells did not express markers for other mesodermal, endodermal and ectodermal lineage tissues (Fig. S3). These results indicate that the combination of CD9, CD140a, CD140b and CD271 can enrich renal progenitor cells differentiated from hiPSCs.

Next, we examined our cell surface marker combination on cells differentiated from 585A1 by using another differentiation method for renal progenitors^12^. In this case, the induction rate of CD9^+^ cells was very low (0.9 ± 0.1%, n = 3) and cells positively stained with the other three surface markers (CD140a, CD140b and CD271) were variable among the experiments (Table S2). Because CD271^+^ cells were less efficiently induced among the CD140a^+^, CD140b^+^ and CD271^+^ cell populations, we isolated the CD140a^+^CD140b^+^ cell population by flow cytometry sorting and confirmed that SIX2^+^ cells were enriched after isolation with these two cell surface markers (Fig. S4).

We previously showed that OSR1^+^SIX2^+^ cells differentiated from hiPSCs by our differentiation method contributed to renal lineage cells and mainly formed 3D proximal renal tubule-like structures, but not glomeruli-like structures, *in vitro*^15^. We also assessed the developmental potential of hiPSC-derived CD9^−^CD140a^+^CD140b^+^CD271^+^ cells isolated on day 28 by a previously reported organ culture procedure^17^ (Fig. S5A). As a result, the aggregates developed tubular structures consisting of cells positive for a proximal renal tubule marker, *Lotus tetragonolobus* lectin (LTL), and for a distal renal tubule marker, E-cadherin (Fig. S5B,C). However, we did not find glomeruli-like structures positively stained for NEPHRIN, PODOCIN, WT1 or PODOCALYXIN in more than 10 examinations (data not shown). These results suggest that CD9^−^CD140a^+^CD140b^+^CD271^+^ cells generated from hiPSCs have similar developmental potential to hiPSC-derived OSR1^+^SIX2^+^ renal progenitors.

### Transplantation of hiPSC-derived CD9^−^CD140a^+^CD140b^+^CD271^+^ renal progenitors ameliorates AKI in mice

**Figure 3:**
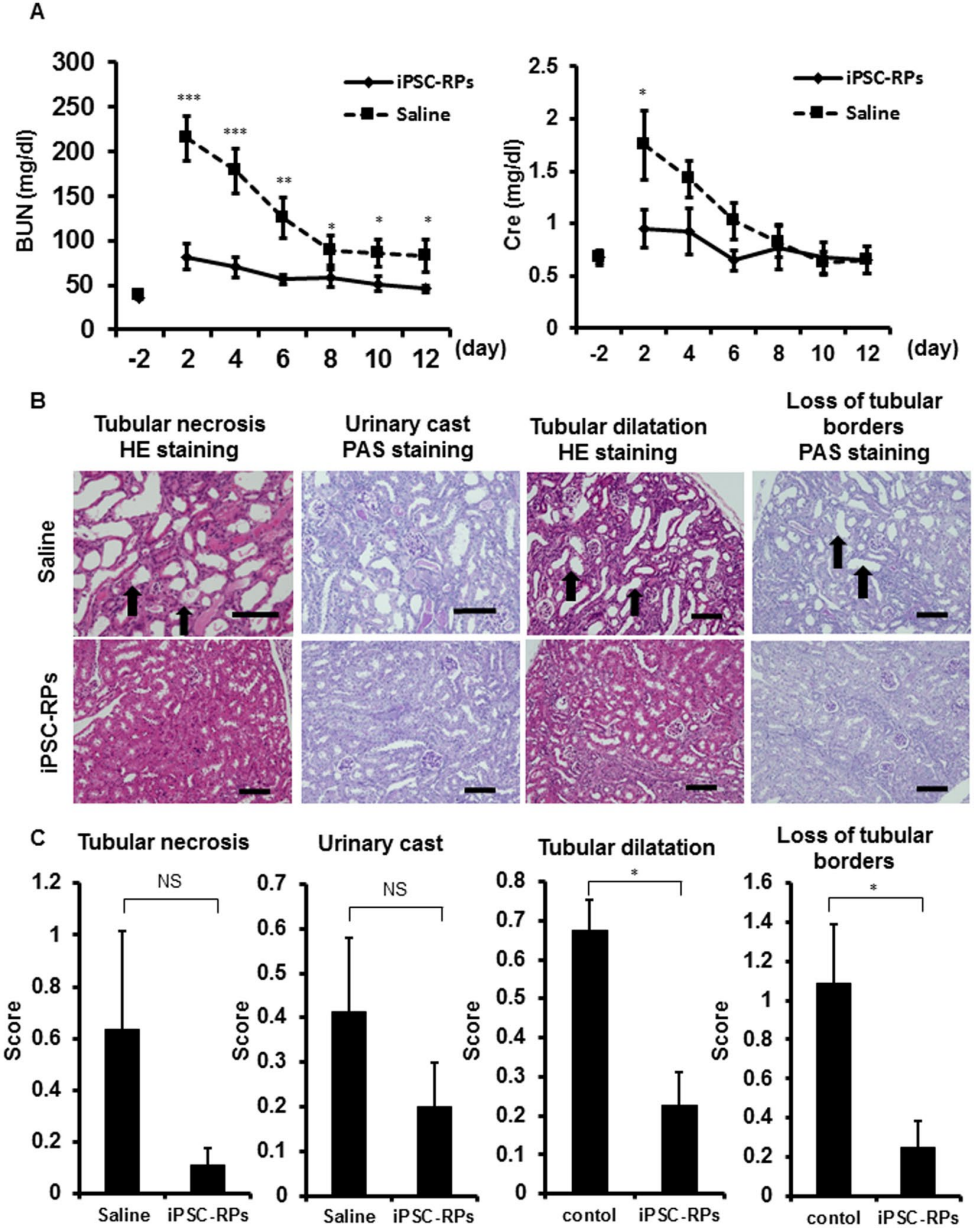
Cell therapy using hiPSC-derived CD9^−^CD140a^+^CD140b^+^CD271^+^ cells for acute kidney injury (AKI) model mice. (**A**) Time course analysis of blood urea nitrogen (BUN, left) and serum creatinine (Cre, right) levels in ischemia/reperfusion (I/R) AKI mice that received a renal subcapsular transplantation of hiPSC-derived CD9^−^CD140a^+^CD140b^+^CD271^+^ cells (n = 4, iPSC-RPs, diamond) or saline injection (n = 4, square). Statistical significance: ***P < 0.001 vs. saline, **P < 0.01 vs. saline and *P < 0.05 vs. saline after multiple testing adjustment. Least square means and 95% confidence intervals were estimated according to the mixed effects model for repeated measures. (**B**) Representative section images of the host mouse kidney samples that received saline injection (upper panels) or transplantation of iPSC-RPs (lower panels) on day 12 after I/R and transplantation. Tubular necrosis, urinary cast, tubular dilatation and loss of tubular borders can be seen in each treatment group. The arrows indicate representative areas of each finding. Scale bars, 100 µm. (**C**) Histological intensity scores of tubular necrosis, urinary cast, tubular dilatation and loss of tubular borders in host kidneys on day 12 after I/R injury (n = 4). Statistical significance: *P < 0.05 vs. saline after multiple testing adjustment. BUN, blood urea nitrogen; Cre, creatinine; HE, Hematoxylin and eosin; PAS, periodic acid-Schiff; NS, not significant.

In order to examine the therapeutic potential of hiPSC-derived renal progenitors isolated with the combination of cell surface markers identified in this study, we transplanted aggregates of hiPSC-derived CD9^−^CD140a^+^CD140b^+^CD271^+^ cells isolated on day 28 into kidney subcapsules of AKI mouse models induced with ischemia reperfusion (I/R) injury just after the injury. A renal functional parameter, blood urea nitrogen (BUN) level, was significantly lower in the mouse group transplanted with hiPSC-derived CD9^−^CD140a^+^CD140b^+^CD271^+^ cells than in the control mouse group injected with saline from 2 to 12 days after I/R. Another parameter, serum creatinine (Cre) level, was also significantly lower in the transplantation group two days after I/R (Fig. 3A). We also compared the therapeutic potential of unsorted cells before isolation and isolated CD9^−^CD140a^+^CD140b^+^CD271^+^ cells, finding that the therapeutic effects of unsorted cells were unstable (Fig. S6). In addition, the serum Cre level on day 2 after I/R was significantly lower when isolated CD9^−^CD140a^+^CD140b^+^CD271^+^ cells were transplanted compared with the unsorted cells. Similarly, the BUN level on day 2 was considerably lower, albeit not significantly, with the transplantation of CD9^−^CD140a^+^CD140b^+^CD271^+^ cells (Fig. S6).

Histological analyses revealed that the areas of kidney injury with tubular dilatation and loss of tubular borders were significantly smaller in the mouse group treated with hiPSC-derived CD9^−^CD140a^+^CD140b^+^CD271^+^ cells than in the saline group (Fig. 3B,C). The other two kidney injury findings, tubular necrosis and urinary cast, were not significantly but considerably smaller in the transplantation group than in the saline group. These results indicate that the transplantation of hiPSC-derived CD9^−^CD140a^+^CD140b^+^CD271^+^ cells ameliorates AKI in mice.

### Transplantation of hiPSC-derived CD9^−^CD140a^+^CD140b^+^CD271^+^ renal progenitors prevents kidney fibrosis after AKI

**Figure 4:**
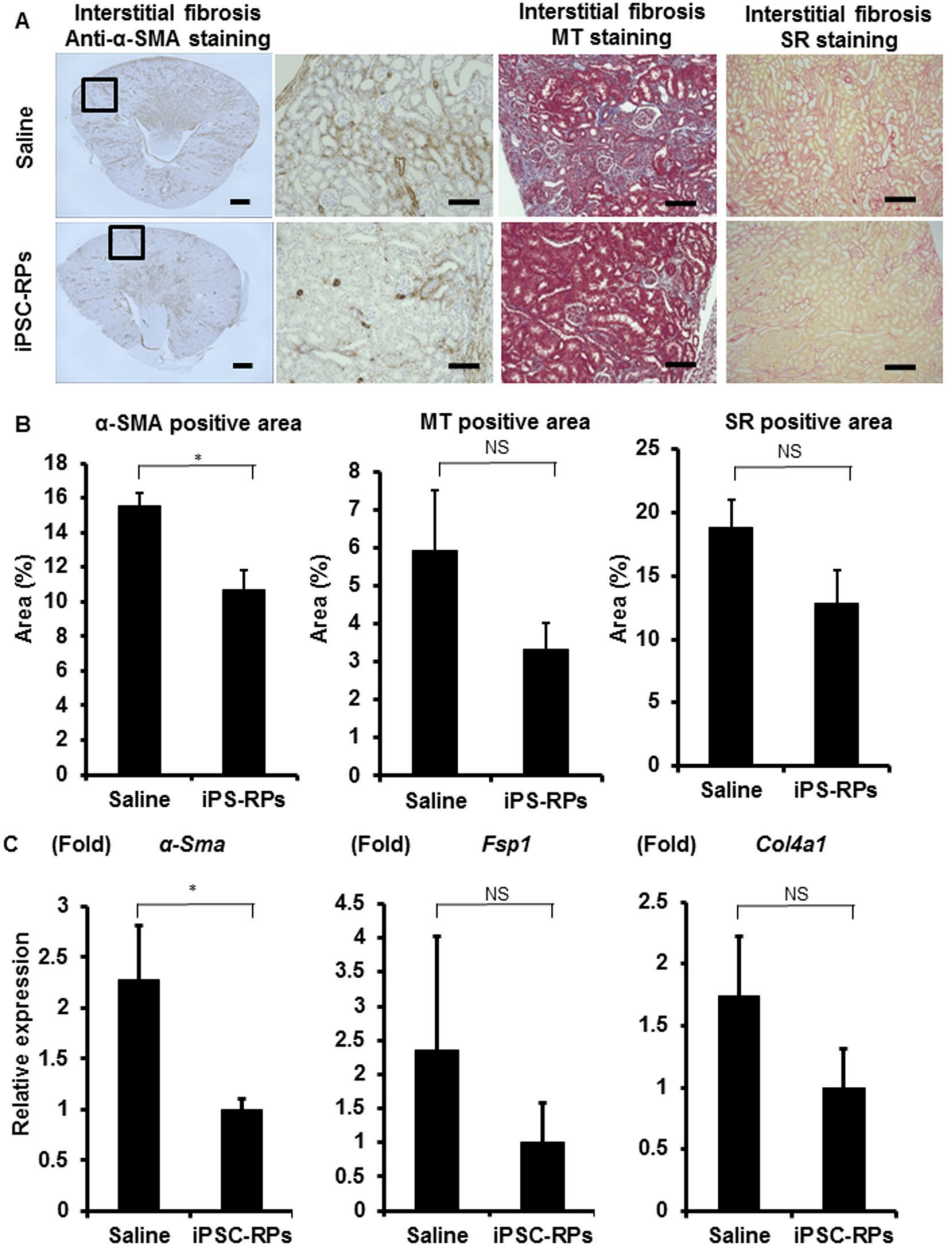
The evaluation of kidney fibrosis after cell therapy using hiPSC-derived CD9^−^CD140a^+^CD140b^+^CD271^+^ cells in mouse acute kidney injury (AKI) models (**A**) Representative images of host mouse kidney sections stained with anti-alpha smooth muscle actin (α-SMA) immunostaining and Masson’s trichrome (MT) and Sirius red (SR) stainings in saline- (upper panels) or CD9^−^CD140a^+^CD140b^+^CD271^+^ cell (iPSC-RP, lower panels)-treated groups. The boxed areas are magnified and displayed in the right panels. Scale bars, 500 µm in the panel to the farthest left and 100 µm in the others. (**B**) Quantitative analyses of kidney fibrosis areas by anti-α-SMA immunostaining and MT and SR stainings in the host kidneys on day 12 after I/R injury (n = 4). Statistical significance: *P < 0.05 vs. saline after multiple testing adjustment. (**C**) qRT-PCR analyses of gene expression in the host kidneys on day 12 after I/R injury for the markers of kidney fibrosis (n = 4). Statistical significance: *P < 0.05 vs. saline after multiple testing adjustment. Fsp1, Fibroblast-specific protein 1; Col4a1, alpha-1 subunit of collagen type IV, NS, not significant.

The interstitial fibrosis occurring after AKI is related to renal prognosis^18^. Histological analysis by anti-α-smooth muscle actin immunostaining revealed that the fibrosis areas were significantly smaller in the transplanted group (Fig. 4A,B), which is consistent with the results of the qRT-PCR analysis for *α-Sma* expression levels (Fig. 4C). The areas of interstitial fibrosis evaluated by Masson’s trichrome and Sirius red stainings as well as the expression levels of other fibrosis markers including *Fsp1* and *Col4a1* were not significantly but considerably smaller in the transplantation group than in the saline group (Fig. 4). These results suggest that the transplantation of hiPSC-derived CD9^−^CD140a^+^CD140b^+^CD271^+^ cells may prevent renal fibrosis after AKI and improve kidney prognosis.

## Discussion

Cell therapy using renal progenitors derived from hESCs/iPSCs is an expected effective treatment for kidney disease. To realize this potential, the removal of contamination by other lineage cells and undifferentiated cells is imperative, as these cells risk neoplastic formations and other unwanted side effects. To solve this problem, the development of an isolation method for renal progenitors by using cell surface markers is required. In the present study, we identified the combination of CD9^−^CD140a^+^CD140b^+^CD271^+^ as most efficient for enriching renal progenitors derived from hiPSCs. Several studies have already reported cell surface markers to isolate clinically useful cell types induced from hESCs/iPSCs, such as cardiomyocytes^19,20^, midbrain dopaminergic progenitor cells^21^, neural stem cells, glia and neurons^22^, pancreatic progenitor cells^23^ and liver progenitor cells^24^. However, cell surface markers for renal progenitors induced from hESCs/iPSCs have not yet been reported. After isolation using the combination of cell surface markers established in this study, we found increased expression levels of renal progenitor markers and decreased marker expressions for other lineages and undifferentiated cells. Furthermore, we demonstrated that CD9^−^CD140a^+^CD140b^+^CD271^+^ cells derived from hiPSCs have therapeutic potential against AKI.

The purity of OSR1^+^SIX2^+^ renal progenitors by our isolation method is not high (63.8 ± 3.3%, n = 3) and should be improved in the future. However, our method can enrich renal progenitors with therapeutic potential on AKI that are differentiated from hESC/iPSC lines without genome editing and can remove any undifferentiated cells. Using our previously reported differentiation protocol^15^, in the present study we generated CD9^−^CD140a^+^CD140b^+^CD271^+^ cells from 585A1 cells at low induction efficiency around day 28. One possible explanation for the efficiency differences is that surface marker expressions could be temporally dependent on the differentiation culture. Another possibility is that the differentiation potentials toward specific lineages are different among hESC/iPSC lines^25–28^. Indeed, Morizane *et al*. demonstrated such substantial differences in the differentiation potential to nephron progenitors^14^. On the other hand, when we differentiated 585A1 cells into nephron progenitors by using another differentiation protocol^12^, the induction rate of CD9^+^ or CD271^+^ cells was very low (Table S2). Different differentiation protocols may require some modifications to acquire renal progenitors of the same character. Future studies are necessary to identify the time course of the purification and the combination of cell surface markers for each cell line or differentiation method.

The surface markers identified in this study were expressed during kidney development. CD140a, also known as platelet-derived growth factor receptor α (PDGFRα), is expressed in metanephric interstitium and vascular arcades that course through the blastema in human fetal kidney^29^. Mice deficient of PDGFRα show a lack of interstitial mesenchyme in the cortex of developing kidney^30^. CD140b, also known as PDGFRβ, is also expressed throughout the interstitium in human fetal kidney^31^. PDGFRβ-expressing cells are located in the cleft of comma- and S-shaped bodies and present a mesangial distribution at later developmental stages^31–33^. PDGFRβ-deficient mice are hemorrhagic, thrombocytopenic and severely anemic, exhibiting an abnormal glomerular phenotype characterized by a total lack of mesangial cells, and die at or shortly before birth^34^. OSR1^+^SIX2^+^ renal progenitors show differentiation potential mainly to renal tubules^15^, while both PDGFRα and PDGFRβ are expressed in the interstitial cells of developing kidneys, which seems an inconsistent finding. Our result is also inconsistent with the findings of a previous report that showed colony-forming renal progenitors can be enriched in the Osr1^+^Itga8^+^Pdgfra^−^ fraction of mouse embryonic kidneys and that the Itga8^+^Pdgfra^−^ fraction contains Six2^+^ cells^12^. One possible explanation for these discrepancies is species differences between humans and mice. Another possibility is the difference in developmental stages. Because both nephron progenitors and interstitial cells develop from MM, OSR1^+^SIX2^+^ cells in the early developmental stage may contain MM cells that express PDGFRα and PDGFRβ. OSR1^+^SIX2^+^ cells induced by our induction protocol may be more immature than the colony-forming renal progenitors reported by Taguchi *et al*.^12^. CD271, also known as nerve growth factor receptor (NGFR), is also expressed in developing kidneys. In rat fetal kidney, Sariola *et al*. demonstrated that NGFR is localized in MM at E13 and that the expression is strong in S-shaped bodies and ceases a few days after birth^35^. They also reported that anti-sense oligonucleotide blockage of NGFR expression inhibited kidney morphogenesis. Li *et al*. recently demonstrated that the SIX2^+^ cell population from human fetal kidneys was enriched in the NGFR^+^EpCAM^−^ fraction^17^. Consistently, we also identified EpCAM (CD326) as a negative selection marker in our screening. CD9, also known as Multidrug resistance-associated protein 1 (MRP1), is expressed in human developing kidneys. In human fetal kidney, Konieczna *et al*. showed that MRP1 expression is negative in the mesenchyme but weakly positive in the metanephric blastema and becomes stronger at the stages of comma- and S-shaped bodies^36^.

Harari-Steinberg *et al*. previously demonstrated that NCAM1 (CD56) can isolate renal progenitor cells from human fetal kidneys^37^. However, in our antibody screening, NCAM1 was not detected as a hit (data not shown). Further, because the differentiation cultures from hESCs/iPSCs contain undifferentiated cells and non-renal lineage cells and because NCAM1 is also expressed in non-renal tissues, such as neural tissues^38^, heart^39^ and skeletal muscle^40^, it may be difficult to use NCAM1 as an isolation marker for renal progenitors differentiated from hESCs/iPSCs.

AKI is directly related to patients’ short-term and long-term morbidity and mortality^41–43^. Especially, kidney fibrosis after AKI is one of the risk factors for progression to chronic kidney disease (CKD)^18^. We found that the hiPSC-derived renal progenitor cells isolated with the combination of cell surface markers established in this study prevented renal fibrosis after AKI induced by I/R injury in mice. One report showed that renal progenitor cells derived from hiPSCs ameliorated AKI induced by cisplatin administration^44^. However, because that study did not isolate renal progenitor cells, the cells transplanted may contain other lineage cells and undifferentiated cells. The isolation method of hiPSC-derived renal progenitor cells without genetic modification of the hiPSCs may give a substantial advantage in future cell therapies and disease modeling using patient-derived hiPSCs. However, we still have some issues to overcome before clinical applications. First, we need to develop long-term expansion cultures to obtain a large number of cells for use in the transplantation therapy, because of the low induction rate of OSR1^+^SIX2^+^ cells from hiPSCs. In addition, we have not yet demonstrated the therapeutic effects of cell therapy using hiPSC-derived renal progenitors for CKD mouse models. Future studies should address these issues toward clinical applications.

In conclusion, we identified the combination of four cell surface markers, CD9^−^CD140a^+^CD140b^+^CD271^+^, that enriches OSR1^+^SIX2^+^ renal progenitor cells at an efficiency above 60%. These isolated renal progenitor cells ameliorated AKI in mice. The isolation method established in this study may provide a tool that contributes to the development of hiPSC-based cell therapy and disease modeling against kidney diseases.

## Materials and Methods

### Cell Culture

The use of hiPSCs was approved by the Ethics Committee of Kyoto University, and informed consent was obtained from all donor subjects from which hiPSC lines were generated in accordance with the Declaration of Helsinki. Cell cultures were performed as previously described^15,45^. Four hiPSC lines: 201B7^46^, 3D45 (an OSR1-GFP knock-in hiPSC line generated from 201B7^45^), 4A6 (an OSR1-GFP/SIX2-tdTomato knock-in hiPSC line generated from 3D45^15^) and 585A1^16^ were maintained on feeder layers of mitomycin C-treated mouse embryonic fibroblasts (Oriental Yeast, Tokyo, Japan) or mouse SNL feeder cells with Primate ES medium (ReproCELL, Kanagawa, Japan) supplemented with 500 U/ml penicillin/streptomycin (PS; Thermo Fisher Scientific, Waltham, MA, USA) and 4 or 5 ng/ml recombinant human basic fibroblast growth factor (FGF; Wako, Osaka, Japan). hiPSCs were passaged at a split ratio of 1:3–1:6 every three to seven days by dissociation with CTK solution consisting of 0.25% trypsin (Thermo Fisher Scientific), 0.1% collagenase IV (Thermo Fisher Scientific), 20% Knockout serum replacement (KSR; Thermo Fisher Scientific) and 1 mM CaCl_2_ in PBS. hiPSCs were routinely examined for mycoplasma contamination.

### Differentiation Protocol

Embryoid body (EB)-based differentiation was performed as previously described^15^. hiPSCs at 70–80% confluency were dissociated with CTK solution, scraped off with a cell scraper and plated into 0.1% or 0.2% gelatin-coated 6 cm dishes with EB medium consisting of Dulbecco’s modified Eagle’s medium (DMEM)/F12 + Glutamax (Thermo Fisher Scientific) supplemented with 0.1 mM non-essential amino acids (Thermo Fisher Scientific), 1,000 U/ml PS, 55 µM 2-mercaptoethanol (Thermo Fisher Scientific) and 20% KSR to remove the feeder cells.

After 1.5–2 h of incubation, the cells were transferred into 35 mm low attachment dishes (SUMITOMO BAKELITE, Tokyo, Japan) or low attachment 6-well plates (Corning, Corning, NY, USA) containing Stage 1 medium (DMEM/F12 + Glutamax supplemented with 500 U/ml PS and 2% fetal bovine serum (FBS, Biosera, Kansas City, MO, USA)) in the presence of 100 ng/ml recombinant human/mouse/rat activin A (R&D Systems, Minneapolis, MN, USA), 1 µM CHIR99021 (Axon Medchem, Groningen, Netherlands) and 10 µM Y-27632 (Abcam Biochemicals, Cambridge, MA, USA) to form EBs. After 48 h from the beginning of the differentiation induction (day 3), EBs were plated into 24-well plates coated with Synthemax II (Corning). The medium was changed to Stage 2 medium (DMEM/F12 + Glutamax containing 0.1 mM non-essential amino acids, 500 U/ml PS, 55 µM 2-mercaptoethanol and 10% KSR) supplemented with 100 ng/ml recombinant human bone morphogenetic protein (BMP)7 (R&D Systems) and 1 µM CHIR99021 to commit the cells to the IM. On day 6, the medium was switched to Stage 2 medium supplemented with 1 µM TTNPB (Santa Cruz Biotechnology, Dallas, TX, USA) and 5 ng/ml transforming growth factor (TGF)-β1 (Peprotech, Rocky Hill, NJ, USA). On day 8, the medium was refreshed with new medium containing the same factors. On day 11, the cells were treated with Stage 2 medium supplemented with 5 ng/ml TGF-β1 and 0.5 µM dorsomorphin homologue (DMH)1 (Tocris Bioscience, Bristol, UK) to induce renal progenitor cells. Subsequently, the medium was replaced every three days.

585A1 cells were differentiated into renal progenitors by modifying another previously reported method^12^. In brief, dissociated hiPSCs were plated into U-shaped bottom low adhesion 96-well plates (SUMITOMO BAKELITE) at a density of 1.0 × 10^4^ cells/well containing DMEM/F12 medium (Thermo Fisher Scientific), 2% B27 without retinoic acid (Thermo Fisher Scientific), 0.1 mM non-essential amino acids, 1,000 U/ml PS, 90 µM 2-mercaptoethanol and 2 mM L-glutamine (Sigma-Aldrich, St. Louis, MO, USA; hereafter called basal medium) supplemented with 10 µM Y-27632 and 0.5 ng/ml BMP4 (R&D Systems). After 24 h (on day 2), the medium was changed to basal medium supplemented with 1 ng/ml recombinant human/mouse/rat activin A and 20 ng/ml recombinant human basic FGF. After 48 h (on day 4), the medium was changed to basal medium supplemented with 1 ng/ml BMP4, 10 µM CHIR99021 and 10 µM Y-27632. On day 10, the aggregates were treated with basal medium supplemented with 10 ng/ml recombinant human/mouse/rat activin A, 3 ng/ml BMP4, 3 µM CHIR99021, 0.1 µM retinoic acid (Sigma-Aldrich) and 10 µM Y-27632. On day 12, the medium was switched to basal medium supplemented with 1 µM CHIR99021, 5 ng/ml FGF9 (Peprotech) and 10 µM Y-27632.

### Antibody Screening

Comprehensive analysis of cell surface markers for the differentiated cells around culture day 28 was performed with BD Lyoplate™ Screening Panels (BD Biosciences, Franklin Lakes, NJ, USA) by flow cytometry. Immunostaining and analysis were performed according to the manufacturer’s protocol.

### Flow Cytometry

The cells were treated with Accumax (Innovative Cell Technologies, San Diego, CA, USA) for 20 min at 37 °C and dissociated by pipetting. Dead cells were distinguished by 7-Amino-Actinomycin D (7AAD; 1:20; TONBO biosciences, Tucson, AZ, USA) or Hoechst 33342 (1:2,000; Thermo Fisher Scientific) and excluded from the analyses. Then, the cells were stained at a concentration of 1.0 × 10^6^ cells/ml with monoclonal antibodies or isotype controls in Brilliant Stain Buffer (BD Biosciences). The cells were analyzed and sorted using a FACS Aria II cell sorter (BD Biosciences). The isolated cells were collected in Stage 2 medium containing 10 µM Y-27632. The data were analyzed using FACS Diva (BD Biosciences) software programs. Details of the antibodies used in this study are shown in Table S3.

### Medium Conditioned by UB Cells (UBCs)

UBC-conditioned medium was generated as previously reported^15^. UBCs (a kind gift from Drs. Sakurai and Barasch)^47^ were cultured with minimal essential media (MEM; Thermo Fisher Scientific) supplemented with 10% FBS. To obtain UBC-conditioned medium, cells at 80% confluency were washed with PBS, and the medium was replaced with Stage 2 medium. After three days of incubation, the conditioned medium was filtered through 0.22 µm filters before use.

### Organ Culture

Tubule-like structures were generated using previously reported methods^15,17^. To form cellular aggregates, CD9^−^CD140a^+^CD140b^+^CD271^+^ cells on day 28 were replated in spindle-shaped bottom low adhesion 96-well plates (SUMITOMO BAKELITE) at a density of 1.0 × 10^5^ cells/well with UBC-conditioned medium supplemented with 50 ng/ml BMP7 and 10 μM Y-27632. After 24 h of incubation (day 2), the medium was changed to UBC-conditioned medium supplemented with 50 ng/ml BMP7, 0.5 μM 6-bromoindirubin-3′-oxime (BIO; Sigma-Aldrich) and 10 μM Y-27632. On day 3, the aggregates were transferred onto transwell inserts (Corning) with KR5 medium (DMEM/F12 + Glutamax containing 0.1 mM non-essential amino acids, 500 U/ml PS, 55 µM 2-mercaptoethanol and 5% KSR) in the presence of 4 µM CHIR99021 and 200 ng/ml FGF2 (Peprotech). After 2 days of culture (day 5), the medium was refreshed with KR5 medium, and the aggregates were cultured for an additional 8 days.

### Transplantation

All animal experiments were approved by the CiRA Animal Experiment Committee and conducted in accordance with the institutional guidelines. The cells for transplantation were prepared using a modified version of a previously reported method^15^. In brief, CD9^−^CD140a^+^CD140b^+^CD271^+^ cells on day 28 isolated by flow cytometry sorting were replated into V-shaped bottom low adhesion 96-well plates (SUMITOMO BAKELITE) at a density of 1.0 × 10^5^ cells/well and treated with the same UBC-conditioned medium containing BMP7, BIO and Y-27632 as described above for 2 days. After washing the cells with saline to remove the media, 16 aggregates (approximately 1.6 × 10^6^ cells) of CD9^−^CD140a^+^CD140b^+^CD271^+^ cells were transplanted into the kidney subcapsules of immunodeficient mice (NOD.CB17-Prkdcscid/J) with AKI.

The mouse AKI models induced by ischemia/reperfusion (I/R) injury were generated as described previously^15,48–50^. Briefly, six-week-old male immunodeficient mice were anaesthetized by isoflurane and maintained at 37 °C. One week after right nephrectomy, the left renal artery was clamped for 31 min with an atraumatic microvascular clamp (Natsume Seisakusho, Osaka, Japan). After removing the clamp, the cellular aggregates were transplanted into the kidney subcapsules, while the control mice received a saline injection.

Peripheral blood tests were performed every two days for blood urea nitrogen (BUN) and serum creatinine (Cre) levels using DRI-CHEM 7000VZ (Fuji film, Tokyo, Japan). Twelve days later, the mice were sacrificed, and kidney tissues were collected for analysis.

### Renal Histopathology

After being removed from the host mice, the kidneys were fixed in 10% neutral buffered formalin and embedded in paraffin. The paraffin blocks were sectioned at 3 µm, and the sections were stained with hematoxylin and eosin (HE), periodic acid-Schiff (PAS), Masson’s trichrome (MT) and Sirius red (SR) stainings. For histological assessments, at least 10 non-overlapping fields in one entire section of each kidney sample were randomly chosen and examined using a 20x objective. The renal morphologic changes were assessed based on well-established methods for AKI^51–53^. Briefly, four changes were examined: 1) tubular necrosis, 2) urinary casts, 3) tubular dilatation and 4) loss of tubular borders on a scale of 0 to 2 (0, none; 1/2, minimal; 1, mild; 1 1/2, moderate; and 2, marked). For objective assessments, these examinations were performed by the CMIC Bioresearch Center Co., Ltd. (Tokyo, Japan). To measure the positive areas by MT and SR stainings and α-SMA immunostaining, 9–16 non-overlapping fields in one entire section of each kidney sample were randomly chosen, and the measurements were performed using BZ analyzer in Hybrid Cell Count mode (Keyence).

### Statistical analysis

Statistics are presented as means ± standard error (SE) or 95% confidence intervals (CI). We applied the student**’s** t test for comparisons between two groups. Changes in BUN and Cre at each time point from baseline are described by the generalized linear mixed model to obtain point estimates and 95% CIs. The model included treatment group, time, and treatment-by-time interaction as factors and random intercept for each subject, and the model parameters were estimated by restricted maximum likelihood. The correlation structure was assumed as unstructured, and the Toeplitz, autoregressive, or compound-symmetry structures were used in order if convergence was not obtained. All statistical analyses were performed using SAS software version 9.4. (SAS Institute Inc, Cary, NC, USA).

## Electronic supplementary material

Supplementary information
