# Author Correction: Development of new method to enrich human iPSC-derived renal progenitors using cell surface markers

**DOI:** 10.1038/s41598-019-46254-0

**Published:** 2019-07-18

**Authors:** Azusa Hoshina, Tatsuya Kawamoto, Shin-Ichi Sueta, Shin-Ichi Mae, Toshikazu Araoka, Hiromi Tanaka, Yasunori Sato, Yukiko Yamagishi, Kenji Osafune

**Affiliations:** 1 Center for iPS Cell Research and Application (CiRA), Kyoto University, Kyoto, Japan; 2 Drug Discovery Research, Astellas Pharma Inc, Tsukuba, Ibaraki, Japan; 3 Department of Global Clinical Research, Graduate School of Medicine, Chiba University, 1-8-1 Inohana, Chuo-ku, Chiba, Japan

Correction to: *Scientific Reports* 10.1038/s41598-018-24714-3, published online 23 April 2018

In Figure 2C, the qRT-PCR data for *SIX2* and *SALL1* were miscalculated. As a result, the graphs of *SIX2* and *SALL1* expression are incorrect. The correct Figure 2 appears below as Figure 1.

**Figure 1:**
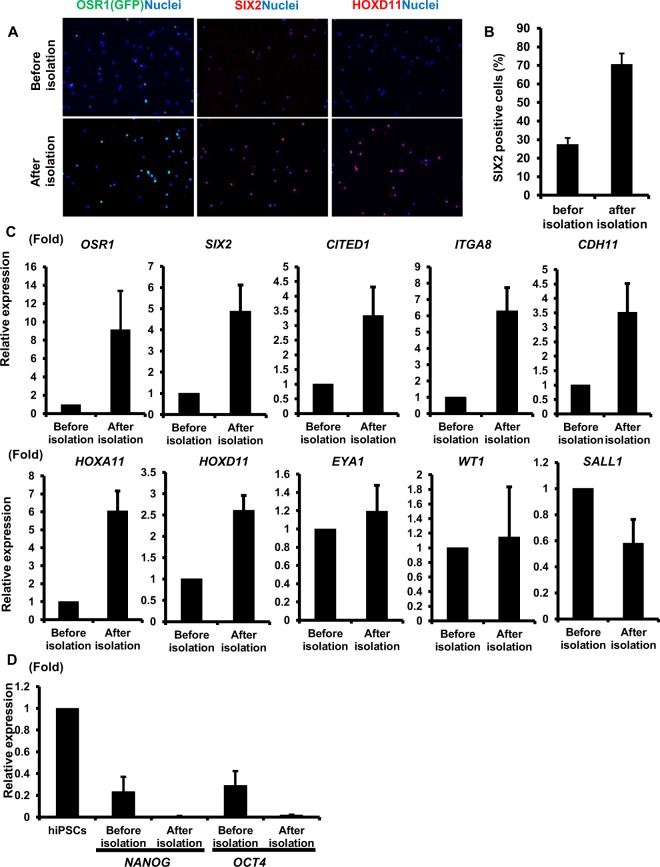
The expression of renal lineage markers in CD9^−^CD140a^+^CD140b^+^CD271^+^ cells isolated from hiPSC differentiation culture. (**A**) Anti-GFP (OSR1), SIX2 and HOXD11 immunostaining images of the cells before isolation (upper panels) and isolated CD9^−^CD140a^+^CD140b^+^CD271^+^ cells (lower panels) on culture days 28 (SIX2) and 30 (OSR1 and HOXD11). Representative images from three independent experiments are shown. Scale bars, 100 μm. (**B**) The induction rates of SIX2+ cells in day 28 cells before and after isolation. The data from three randomly chosen fields are presented as the mean ± SE (n = 3). (**C**,**D**) qRT-PCR analyses of gene expressions in day 28 cells before and after isolation for markers of nephron progenitors (**C**) and undifferentiated cells (**D**). The data from three independent experiments are presented as the mean ± SE (n = 3). GAPDH was used as an internal control, and the relative expression levels were normalized to those of day 28 cells before isolation (**C**) and hiPSCs (**D**). ITGA8, integrin alpha 8; CDH11, cadherin 11.

Additionally in Figure 4C, the qRT-PCR data for *Col4a1* were also miscalculated. As a result, the graph of *Col4a1* expression is incorrect. The correct Figure 4 appears below as Figure 2.

**Figure 2:**
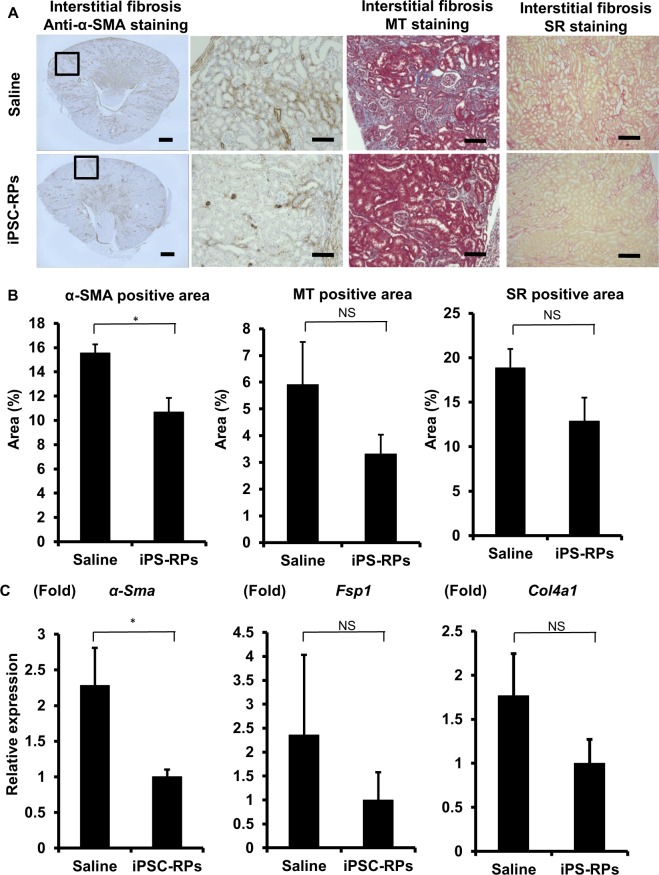
The evaluation of kidney fibrosis after cell therapy using hiPSC-derived CD9^−^CD140a^+^CD140b^+^CD271^+^cells in mouse acute kidney injury (AKI) models (**A**) Representative images of host mouse kidney sections stained with anti-alpha smooth muscle actin (α-SMA) immunostaining and Masson’s trichrome (MT) and Sirius red (SR) stainings in saline- (upper panels) or CD9^−^CD140a^+^CD140b^+^CD271^+^ cell (iPSC-RP, lower panels)-treated groups. The boxed areas are magnified and displayed in the right panels. Scale bars, 500 μm in the panel to the farthest left and 100 μm in the others. (**B**) Quantitative analyses of kidney fibrosis areas by anti-α-SMA immunostaining and MT and SR stainings in the host kidneys on day 12 after I/R injury (n = 4). Statistical significance: *P < 0.05 vs. saline after multiple testing adjustment. (**C**) qRT-PCR analyses of gene expression in the host kidneys on day 12 after I/R injury for the markers of kidney fibrosis (n = 4). Statistical significance: *P < 0.05 vs. saline after multiple testing adjustment. Fsp1, Fibroblast-specific protein 1; Col4a1, alpha-1 subunit of collagen type IV, NS, not significant.

