# Supplementary material for: Development of new method to enrich human iPSC-derived renal progenitors using cell surface markers
Source: Sci Rep. 2018 Apr 23;8:6375. doi: 10.1038/s41598-018-24714-3 (PMC5913312; doi:10.1038/s41598-018-24714-3)
Supplement: Supplementary file 1 — Supplementary information [file 41598_2018_24714_MOESM1_ESM.pdf]

Supplementary information

Development of new method to enrich human iPSC-derived renal progenitors using cell surface markers

Azusa Hoshina[1], Tatsuya Kawamoto[2], Shin-Ichi Sueta[1], Shin-Ichi Mae[1], Toshikazu Araoka[1], Hiromi Tanaka[1], Yasunori Sato[3], Yukiko Yamagishi[2], Kenji Osafune[1]*

[1]Center for iPS Cell Research and Application (CiRA), Kyoto University, Kyoto, Japan

[2]Drug Discovery Research, Astellas Pharma Inc, Tsukuba, Ibaraki, Japan

[3]Department of Global Clinical Research, Graduate School of Medicine, Chiba University, 1-8-1 Inohana, Chuo-ku, Chiba, Japan

***Corresponding author**

Kenji Osafune. Address: 53 Kawahara-cho, Shogoin, Sakyo-ku, Kyoto 606-8507, Japan. E-mail address: osafu@cira.kyoto-u.ac.jp

Tatsuya Kawamoto. Address: 21 Miyukigaoka, Tsukuba, Ibaraki 305-8585, Japan.

E-mail address: tatsuya.kawamoto@astellas.com

Fig. S1

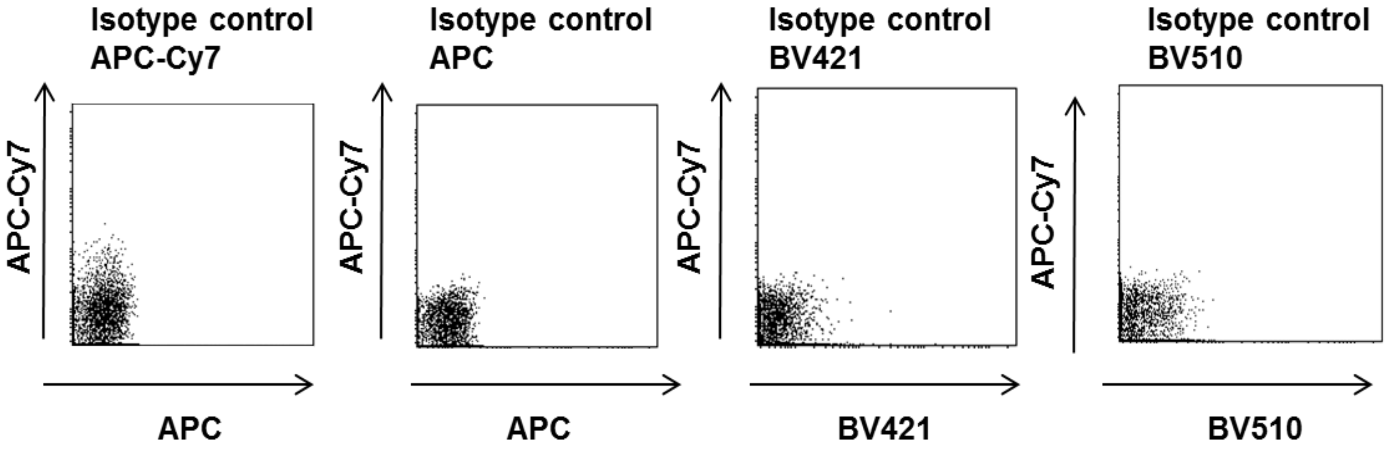

Figure S1. Flow cytometric analysis using isotype controls, related to Fig. 1E.
Flow cytometric analysis using IgG isotypes corresponding to each surface marker.

Fig. S2

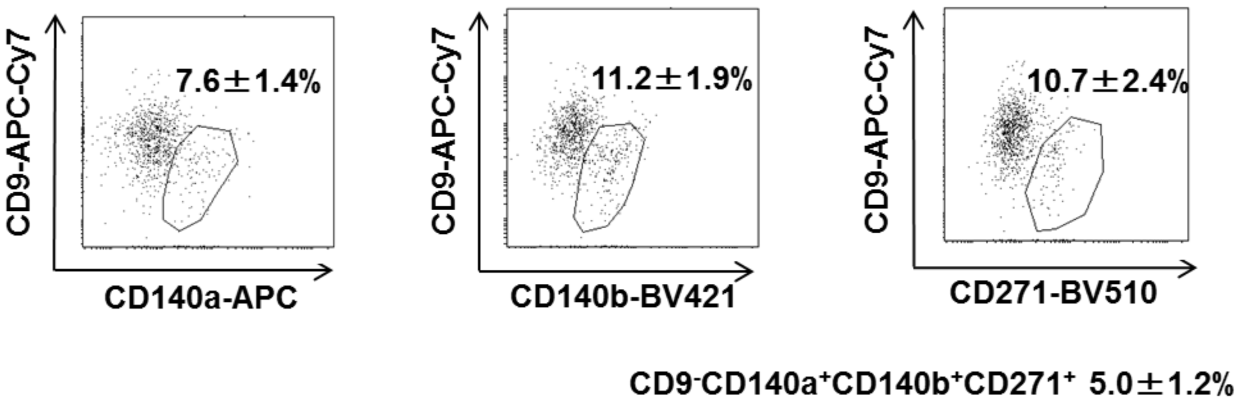

Figure S2. Flow cytometric analysis using cell surface markers on 585A1 cells.
Differentiated cells fractioned with antibodies directed against CD9, CD140a, CD140b and CD271 on culture days 28-30. Representative data from at least three independent experiments are shown and are presented as the mean ± SE (n=3).

Fig. S3

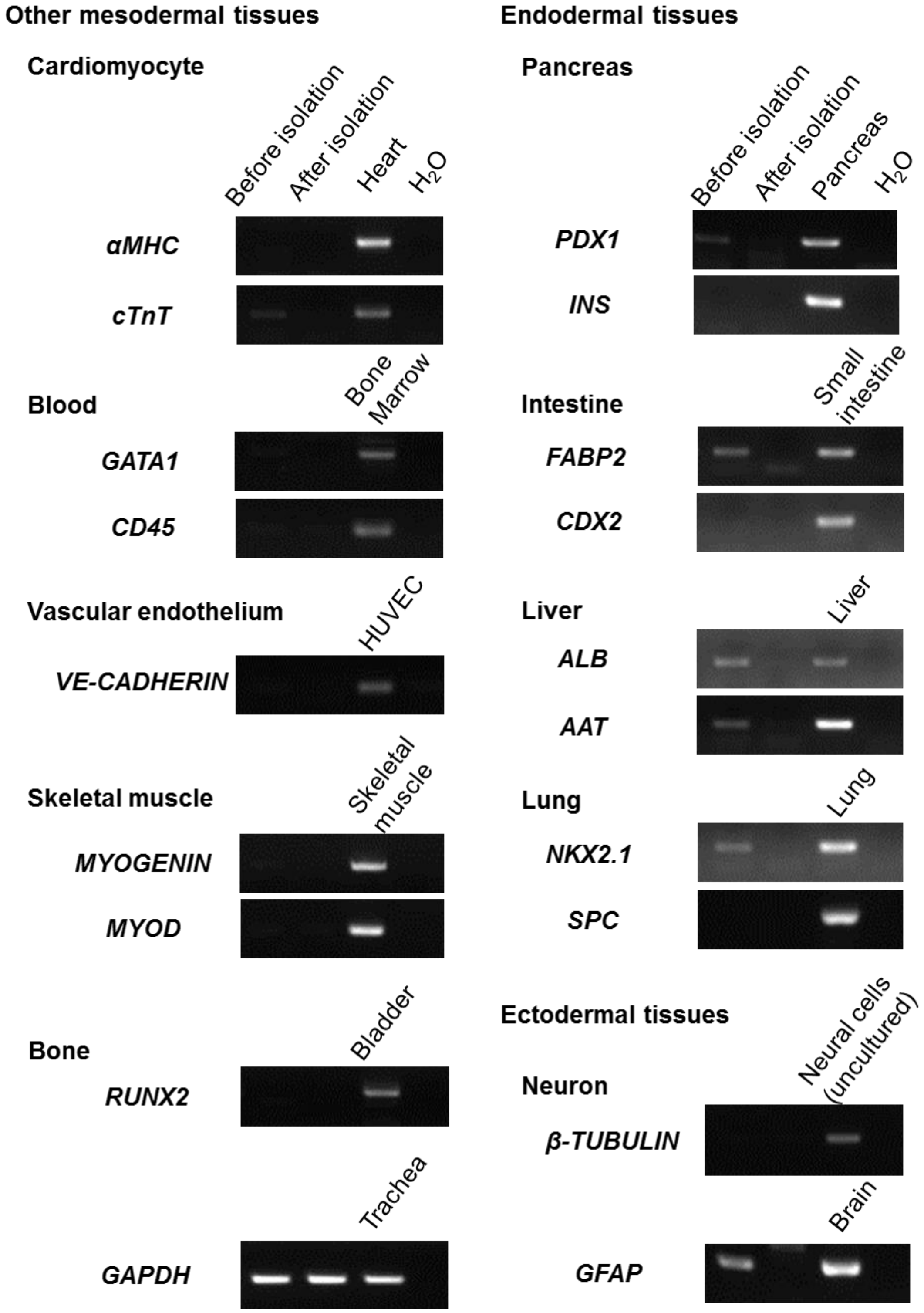

Figure S3. The expression of other lineage markers in CD9$^-$CD140a$^+$CD140b$^+$CD271$^+$ cells isolated from hiPSC differentiated cultures.

The gene expressions of markers for other mesodermal, endodermal and ectodermal lineages in cells before isolation and CD9$^-$CD140a$^+$CD140b$^+$CD271$^+$ cells isolated on day 28 as analyzed by RT-PCR. αMHC, alpha-myosin heavy chain; cTNT, cardiac troponin T; HUVEC, human umbilical vein endothelial cell; INS, insulin; FABP2, fatty acid binding protein; ALB, albumin; AAT, alpha-1 antitrypsin; SPC, surfactant protein C; GFAP, glial fibrillary acidic protein.

Fig. S4

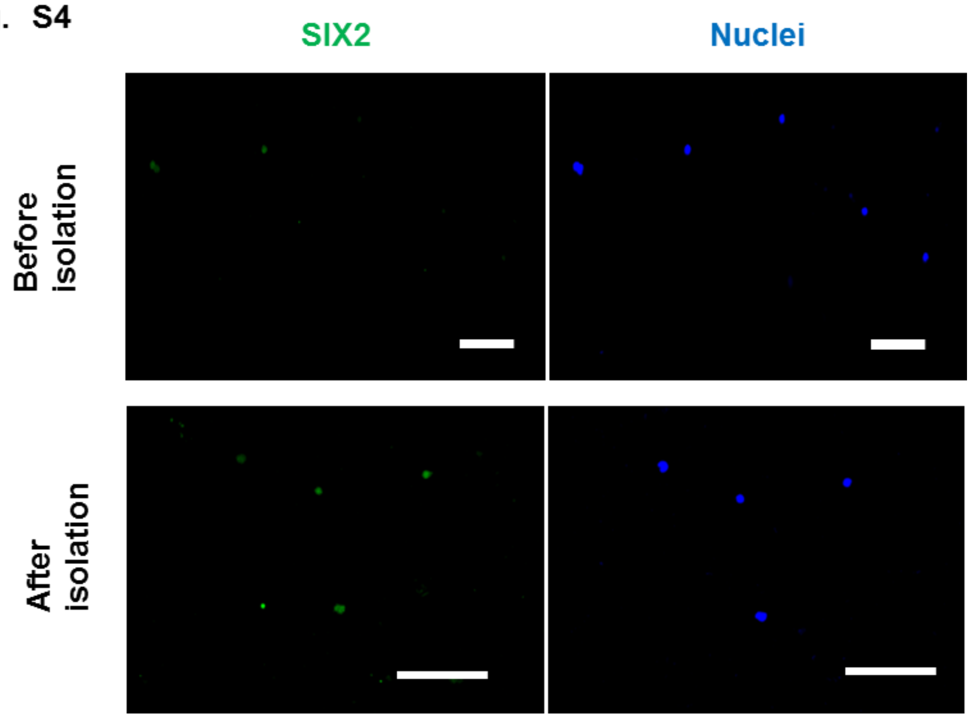

Figure S4. The expression of SIX2 in CD140a$^+$CD140b$^+$ cells isolated from hiPSC differentiation culture by a previously reported method[12].

Anti-SIX2 immunostaining images of the cells before isolation (upper panels) and isolated CD140a$^+$CD140b$^+$ cells (lower panels) on culture day 15. Scale bars, 100 µm. Representative data from at least three independent experiments are shown.

Fig. S5

A

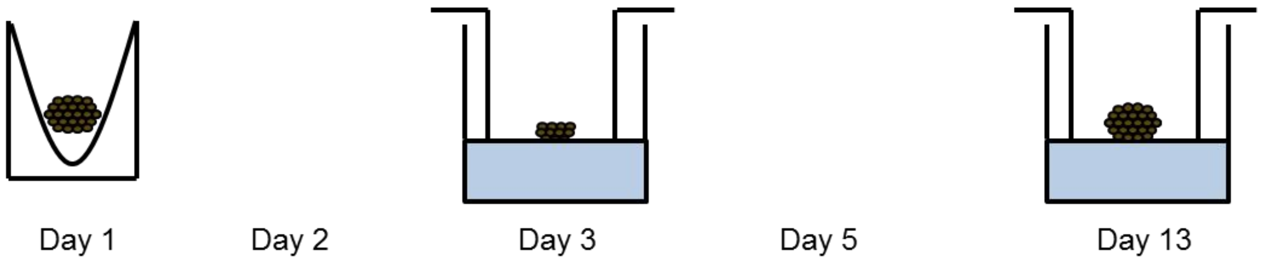

BMP7 (50 ng/ml)	BMP7 (50 ng/ml)	CHIR99021 (4 μM)	
Y-27632 (10 μM)	BIO (0.5 μM)	FGF2 (200 ng/ml)	
	Y-27632 (10μM)		
UBC conditioned medium		5% knockout serum replacement	

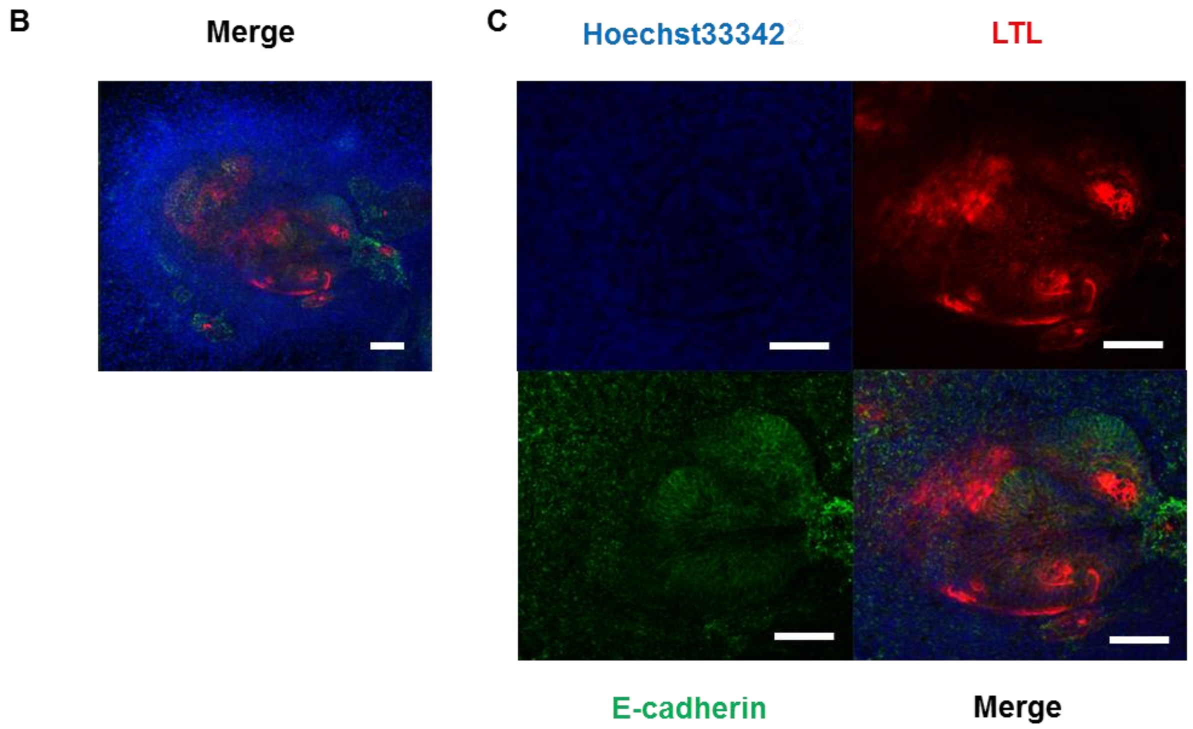

Figure S5. Developmental potential of hiPSC-derived CD9^{-}CD140a^{+}CD140b^{+}CD271^{+} cells to differentiate into renal lineage cells.

(A) A schematic showing cell aggregate cultures in an organ culture setting. (B, C) Images of *Lotus tetragonolobus* lectin (LTL) and anti-E-cadherin antibody stainings of CD9^{-}CD140a^{+}CD140b^{+}CD271^{+} cell aggregates after 10 days of organ culture. Panels in (C) are magnified views of (B) with individual staining or merged staining. Representative data from at least three independent experiments are shown in (B) and (C). Scale bars, 100 μm.

Fig. S6

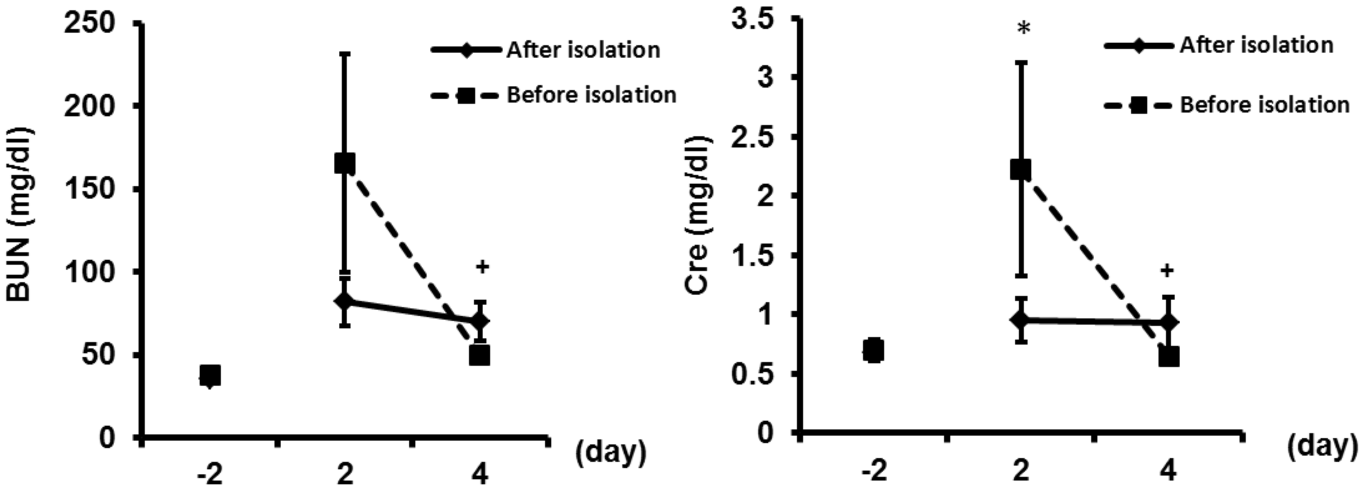

Figure S6. Therapeutic effects of cell therapy for acute kidney injury (AKI) model mice using differentiated cells before isolation or isolated $CD9^{-}CD140a^{+}CD140b^{+}CD271^{+}$ cells. Time course analysis of blood urea nitrogen (BUN, left) and serum creatinine (Cre, right) levels in ischemia/reperfusion (I/R) AKI mice that received a renal subcapsular transplantation of hiPSC-derived $CD9^{-}CD140a^{+}CD140b^{+}CD271^{+}$ cells (n=4, after isolation, diamond) or differentiated cells before isolation (n=4, square). Statistical significance: *P<0.05 vs. before isolation after multiple testing adjustment. Least square means and 95% confidence intervals were estimated according to the mixed effects model for repeated measures. +, Two mice out of four transplanted with cells before isolation died between days 2 to 4 after I/R and transplantation.

Full unedited gel for Figure S3

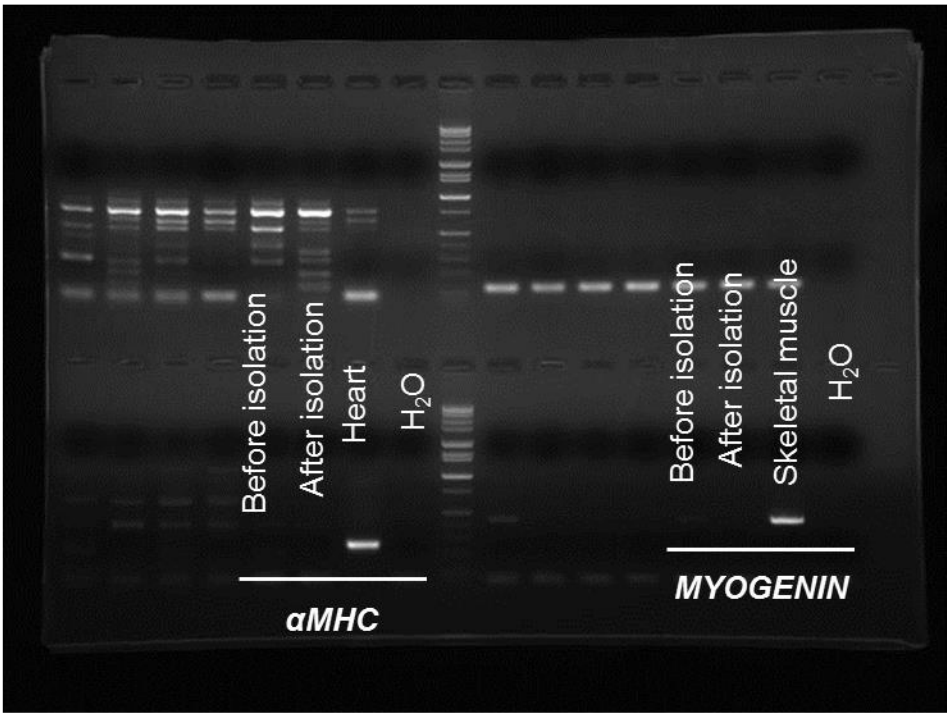

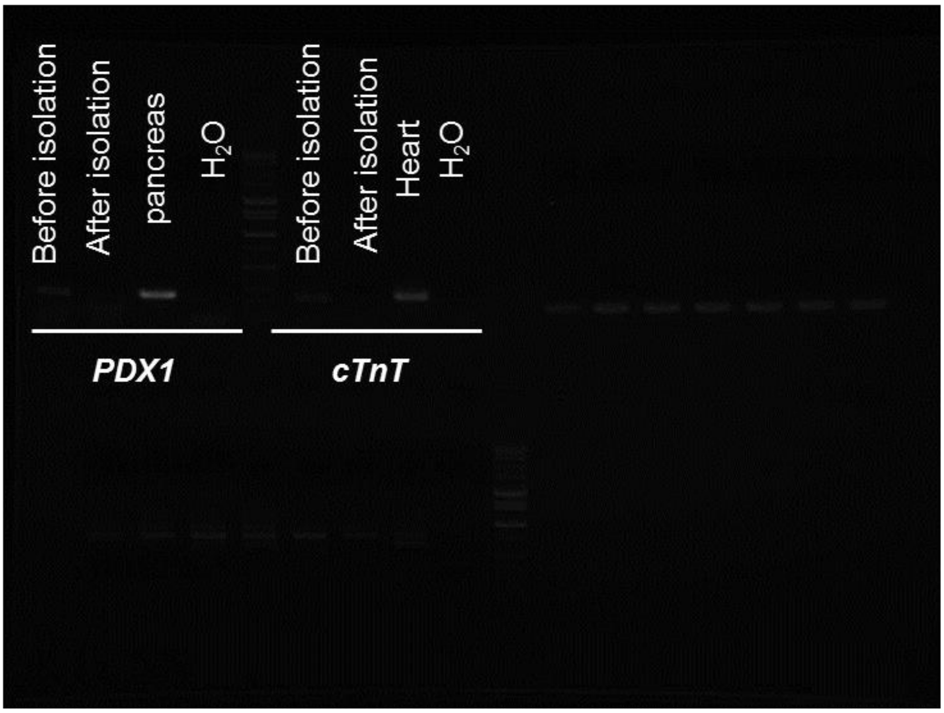

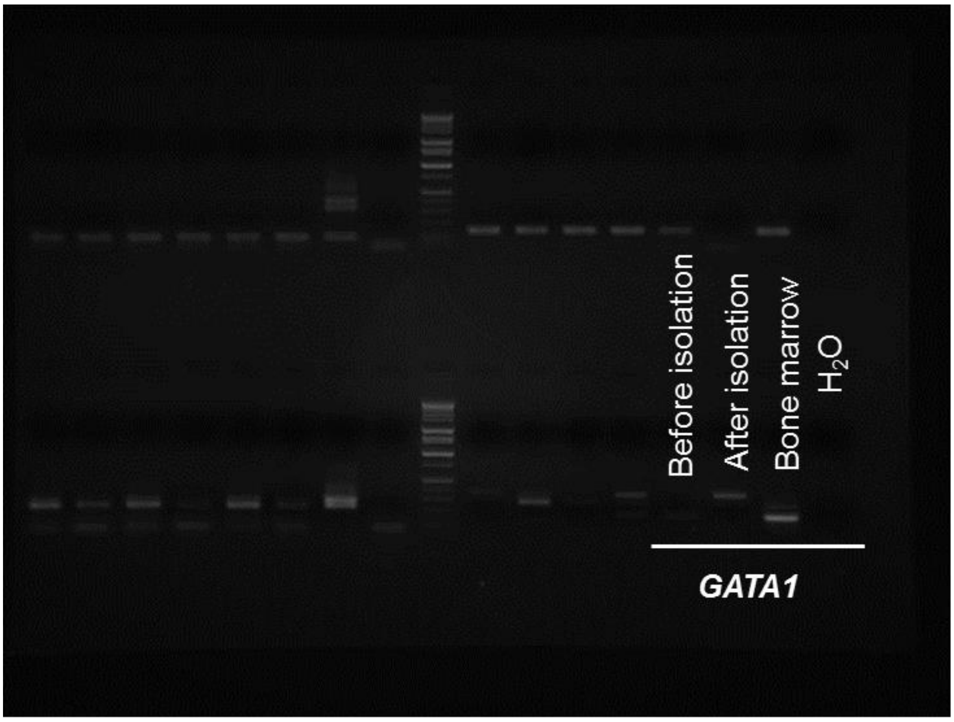
Before isolation
After isolation
Bone marrow
H_2O
GATA1

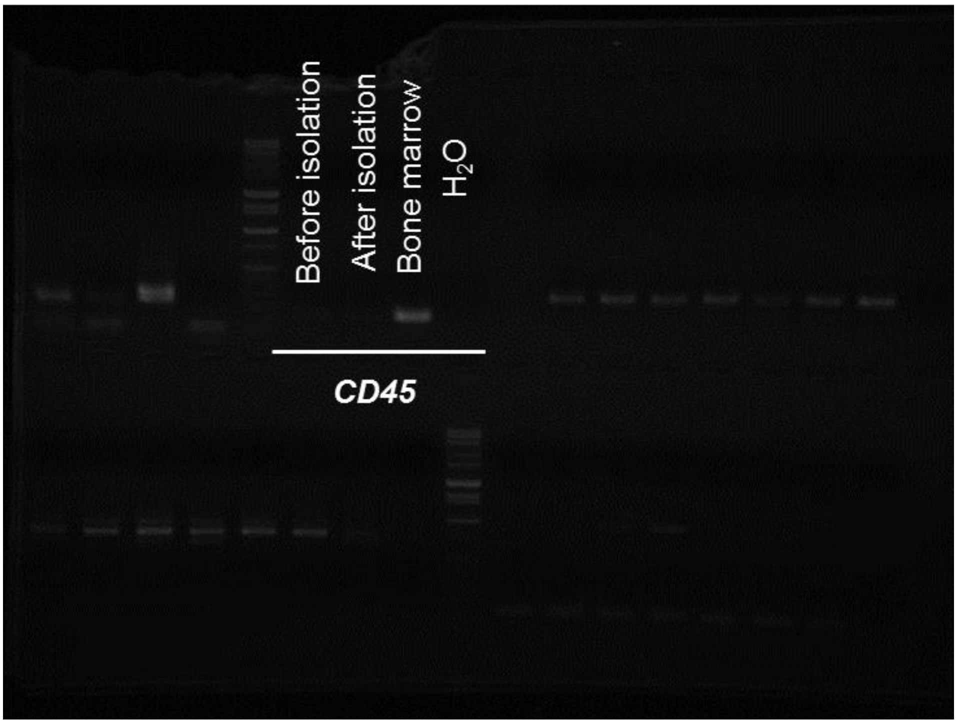
Before isolation
After isolation
Bone marrow
H_2O
CD45

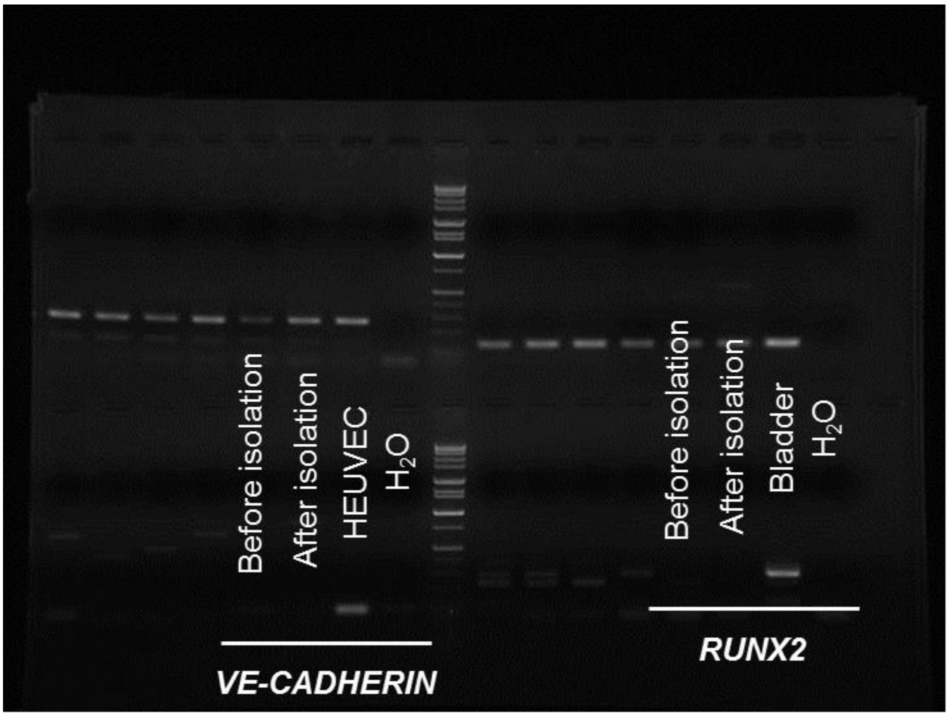
Before isolation
After isolation
HEUVEC
H_2O
VE-CADHERIN
Before isolation
After isolation
Bladder
H_2O
RUNX2

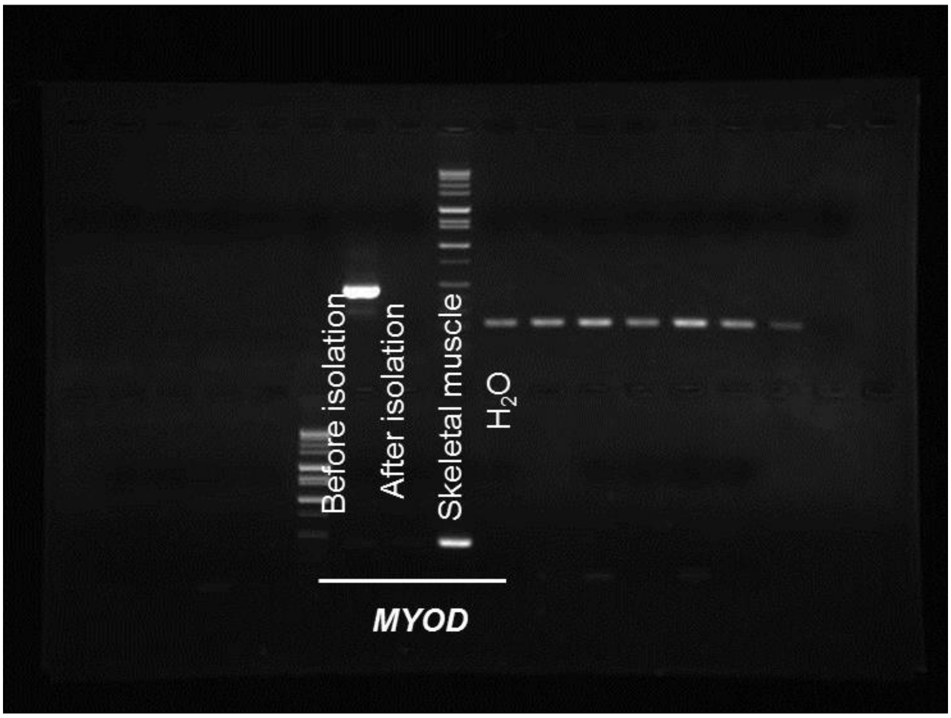
Before isolation
After isolation
Skeletal muscle
H_2O
MYOD

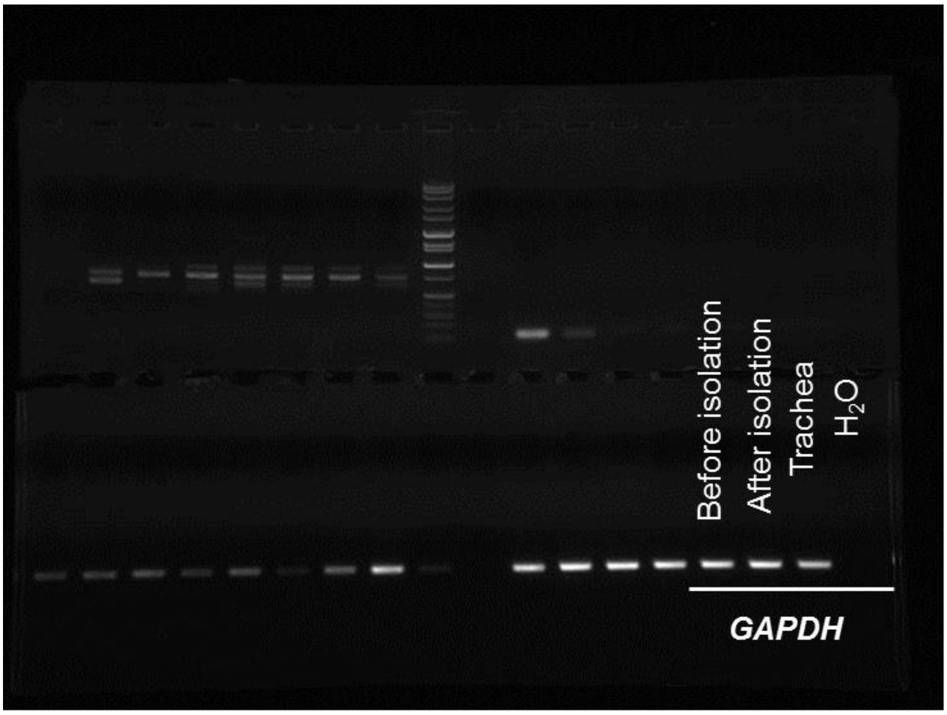
Before isolation
After isolation
Trachea
H_2O
GAPDH

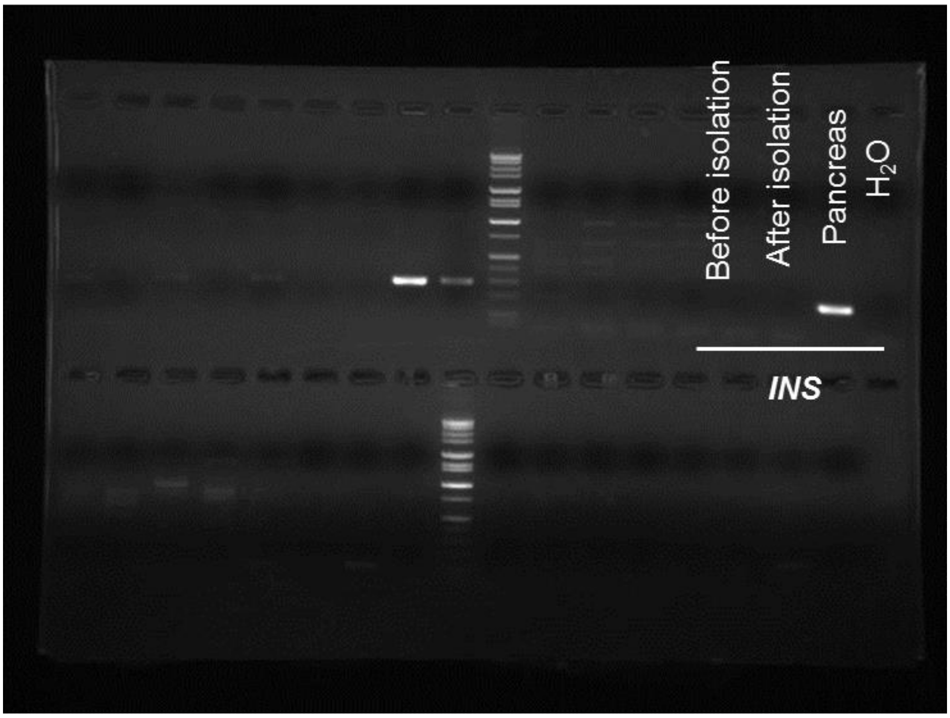
Before isolation
After isolation
Pancreas
H_2O
INS

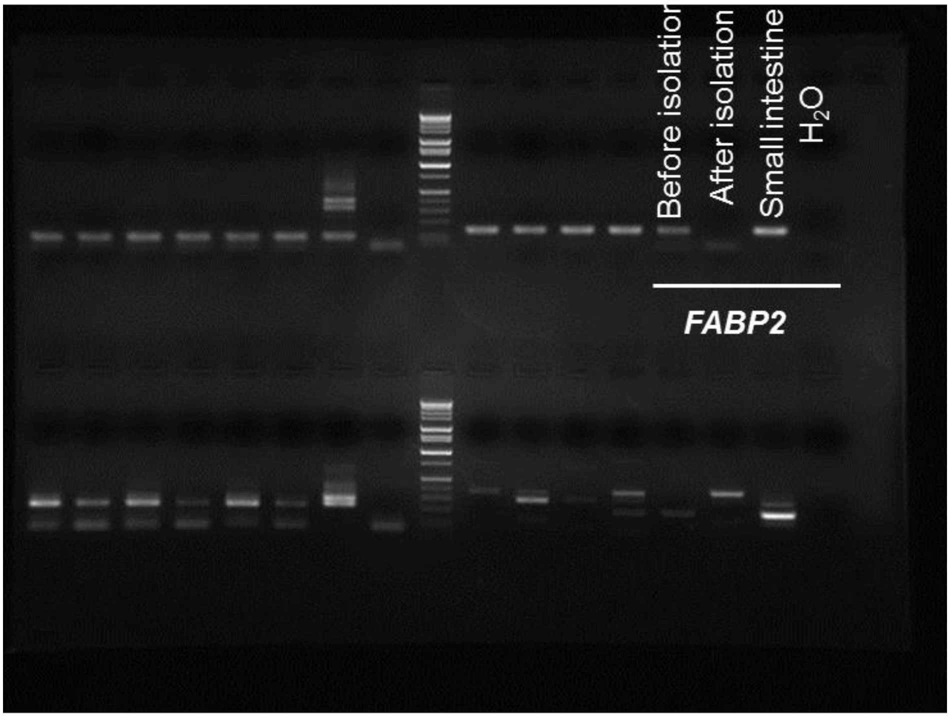
Before isolation
After isolation
Small intestine
H_2O
FABP2

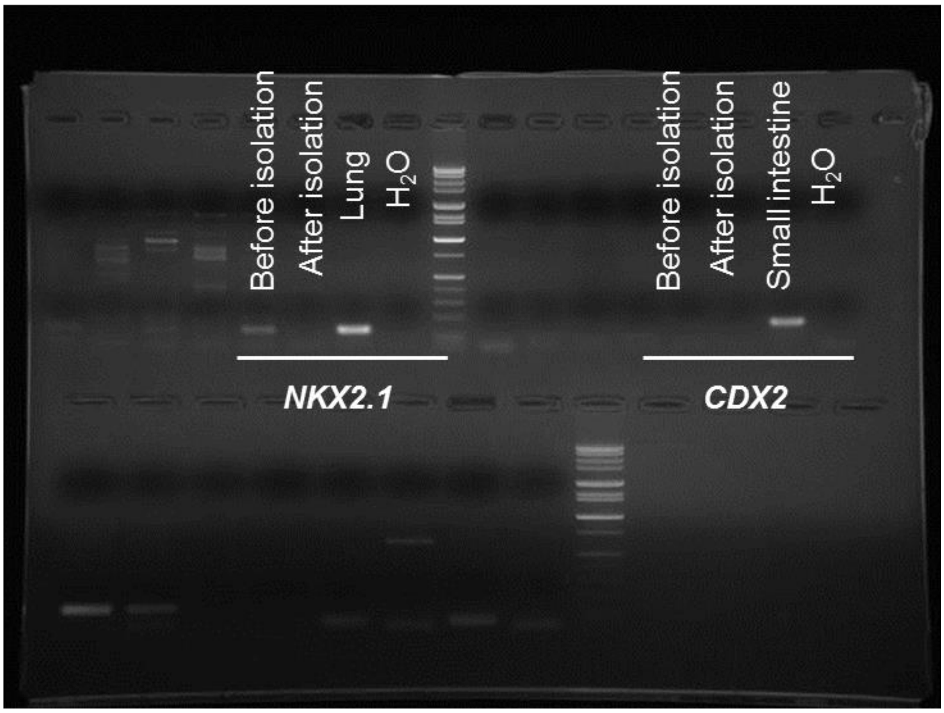
Before isolation
After isolation
Lung
H_2O
NKX2.1
Before isolation
After isolation
Small intestine
H_2O
CDX2

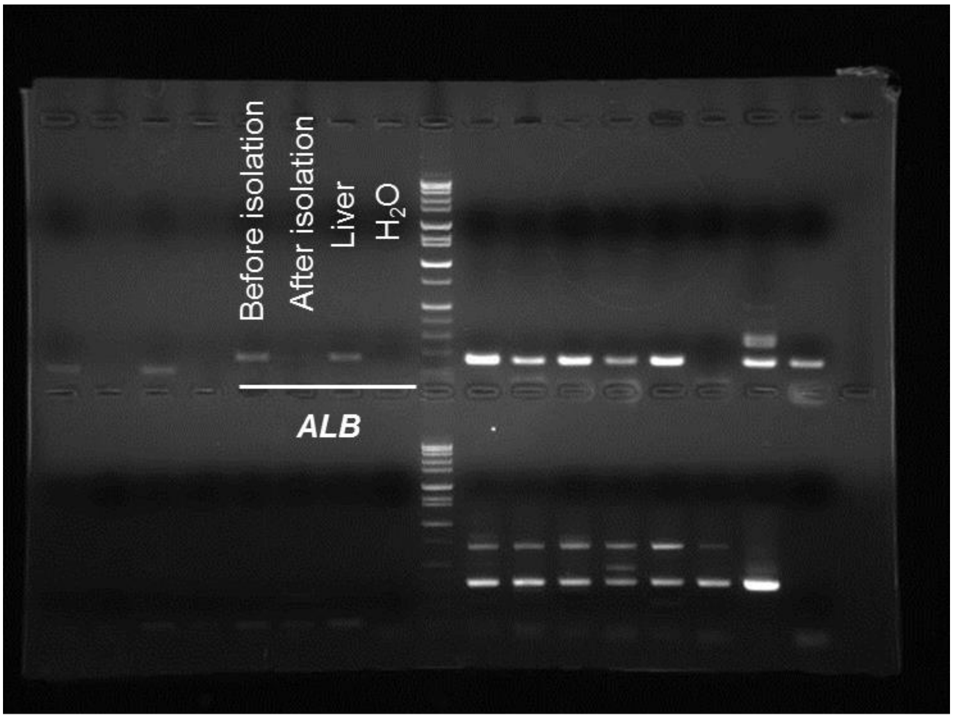
Before isolation
After isolation
Liver
H_2O
ALB

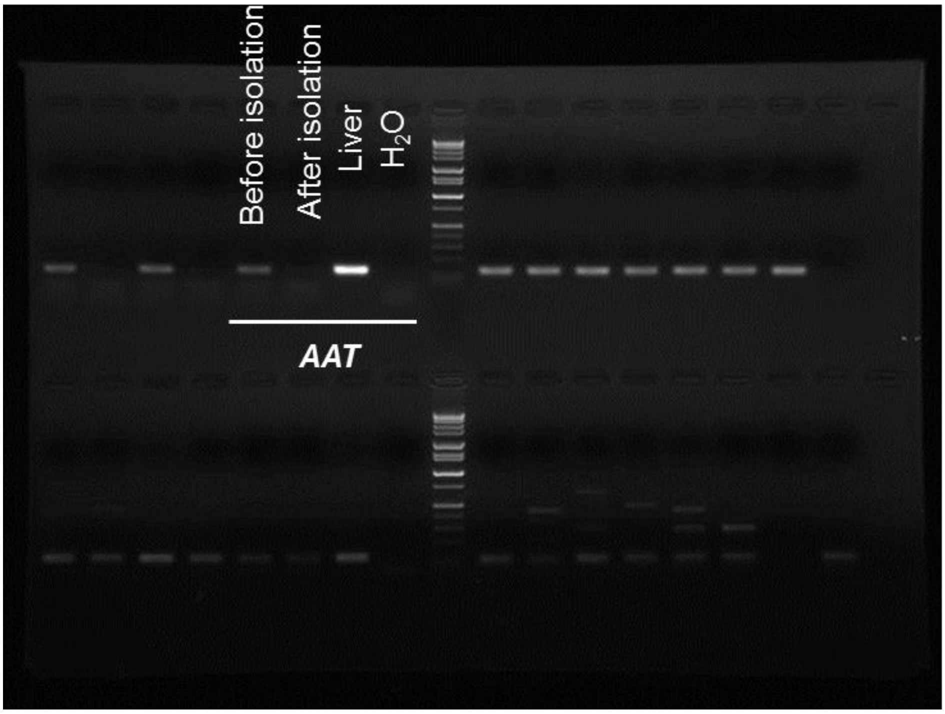
Before isolation
After isolation
Liver
H_2O
AAT

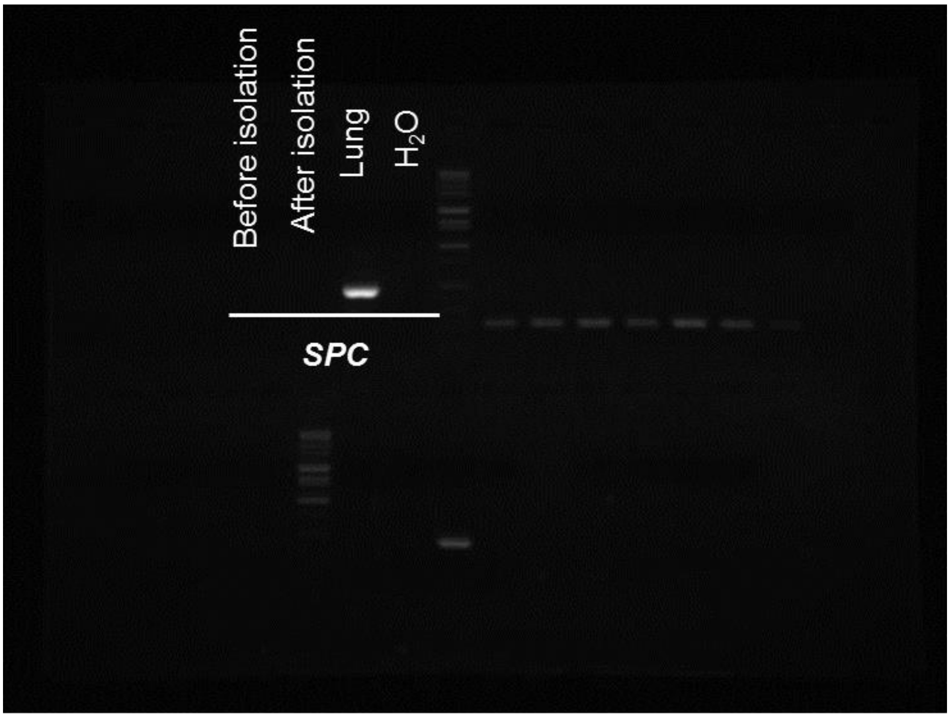
Before isolation
After isolation
Lung
H_2O
SPC

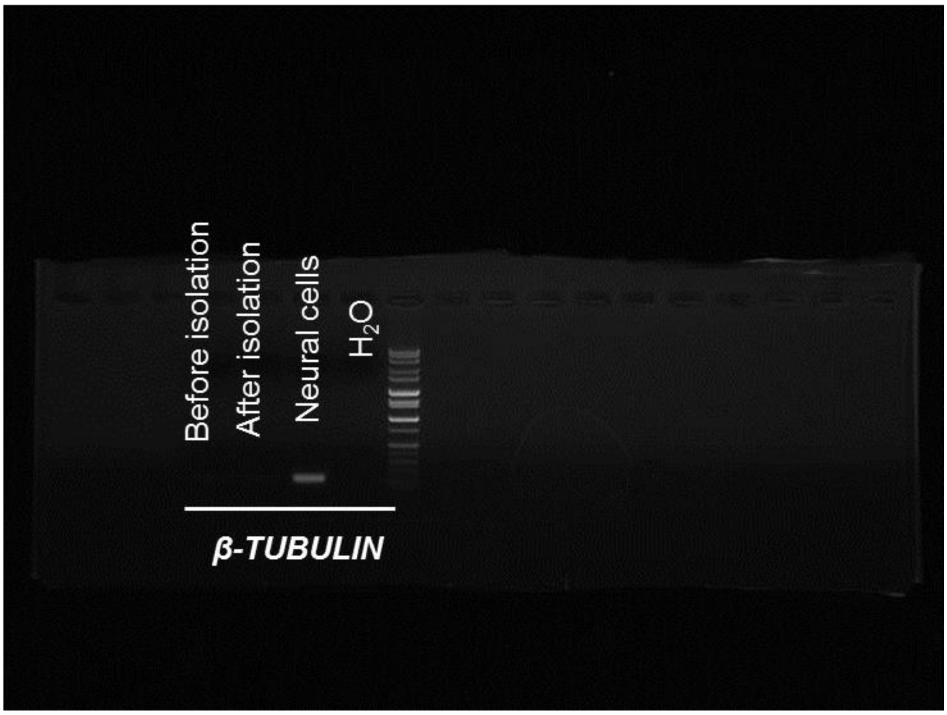
Before isolation
After isolation
Neural cells
H_2O
β-TUBULIN

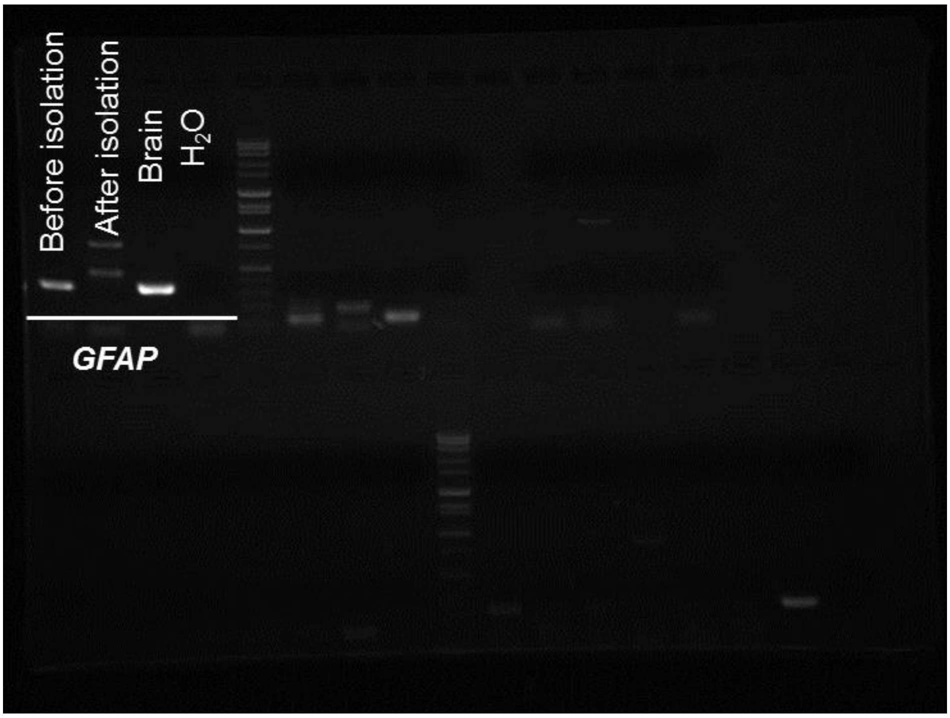
Before isolation
After isolation
Brain
H_2O
GFAP

Supplemental Tables

Table S1. Efficiency of obtaining $OSR1^+SIX2^+$ cells using various combinations of cell surface markers.

Combination of surface marker	Induction rate of $OSR1^+SIX2^+$cells (%)	Ratio of $OSR1^+SIX2^+$cells obtained with each combination (%)
$CD9^-CD140a^+$	40.2	68.4
$CD9^-CD140b^+$	40.2	63.4
$CD9^-CD271^+$	40.2	68.5
$CD9^-CD140a^+CD140b^+$	40.2	69.1
$CD9^-CD140a^+CD271^+$	40.2	73.9
$CD9^-CD140b^+CD271^+$	40.2	71.3
$CD9^-CD140a^+CD140b^+CD271^+$	40.2	74.0
$CD140a^+CD140b^+CD271^+$	40.2	72.4

Table. S2 The induction rate of cells positive for each cell surface marker in differentiation culture by a previously reported method[12].

Experiment	$CD9^+$cells (%)	$CD140a^+$cells (%)	$CD140b^+$cells (%)	$CD271^+$cells (%)
No.1	0.9	95.1	25	5.4
No.2	1.0	83.4	91.5	42.1
No.3	0.7	30.2	43.9	1.0
Mean ± SE	0.9 ± 0.1	69.6 ± 20.0	53.5 ± 19.8	16.2 ± 13.0

Table S3. The antibodies used for flow cytometry in this study.

Antibody	Dilution rate	Manufacturer
CD9 APC-H7	1:20	BD Biosciences, clone M-L13,655409
Alexa Fluor® 647 Mouse anti-Human CD140a	1:20	BD Biosciences, 562798
BV421 Mouse anti-Human CD140b	1:20	BD Biosciences, 564124
BV510 Mouse anti-Human CD271	1:20	BD Biosciences, 563451
APC-H7 Mouse IgG1κ	1:20	BD Biosciences, 560167
APC Mouse IgG1κ	1:20	BD Biosciences, 550854
BV421 Mouse IgG2aκ	1:20	BD Biosciences, 562439
BV510 Mouse IgG2aκ	1:20	BD Biosciences, 562946
PE Mouse anti-Human CD55	1:20	BD Biosciences, 555694
APC Mouse anti-Human CD55	1:20	BD Biosciences, 555696
APC Mouse anti-Human CD326	1:20	Miltenyl Biotec, 130-091-254

Table S4. The primer sets used in this study.

Gene name	Primer Sequence, (5'-3')
hOSR1	GCTGTCCACAAGACGCTACA
	CCAGAGTCAGGCTTCTGGTC
hSIX2	AGGAAAGGGAGAACAACGAGAA
	GGGCTGGATGATGAGTGGT
hCITED1	AGGATGCCAACCAAGAGATG
	TGGTTCCATTTGAGGCTACC
hITGA8	CTGTCAGGCGTTCAACC
	CACCAAGACACTCGCTGTG
hCDH11	CAGTCGGCAGAAGCAGG
	TTGATGGTGAGGGTGTTGG
hHOXA11	CGAAGTGACCTTCAGAGAGT
	ACGGTGCTATAGAAATTGGA
hHOXD11	TGGAACGCGAGTTTTTCTTT
	CTGCAGACGGTCTCTGTTCA
hEYA1	CTACTCAGCTCATCCCAGCATT
	CATACACCACTTTTCTTCCAAACCT
hWT1	GGCAGCACAGTGTGTGAACT
	CCAGGCACACCTGGTAGTTT
hSALL1	AGCGAAGCCTCAACATTTCCAATCC
	AATTCAAAGAACTCGGCACAGCACC
hNANOG	GATTTGTGGGCCTGAAGAAA
	TTGGGACTGGTGGAAGAATC
hOCT4	TGGGCTCGAGAAGGATGTG
	GCATAGTCGCTGCTTGATCG
hGAPDH	GAAGGTGAAGGTCGGAGTC
	GAAGATGGTGATGGGATTTC
hαMHC	CTGGAGGCCGAGCAGAAGCGCAACG
	GTCCGCCCGCTCCTCTGCCTCATCC
hcTnT	ATGAGCGGGAGAAGGAGCGGCAGAAC
	TCAATGGCCAGCACCTTCCTCCTCTC
hGATA1	CAAGCTTCGTGGAACTCTCC
	ACTGACAATCAGGCGCTTCT
CD45	TGTGATGCTTGTTCCCTTCA
	ACTGGAGTGTGGAGCAGCTT
hVE-CADHERIN	ACACCTCACTTCCCCATCA
	GACCTTGCCCACATATTCTCC

hMYOGENIN	TAAGGTGTGTAAGAGGAAGTCG
	CCACAGACACATCTTCCACTGT
hMYOD	CGATATACCAGGTGCTCTGAGGG
	GGGTGGGTTACGGTTACACCTGC
hRUNX2	ATGCTTCATTCGCCTCACAAACAAC
	TGAAGCGCCGGCTGGTGCTC
hPDX1	TGTTCCGAGGTAGAGGCTGT
	AACATAACCCGAGCACAAGG
hINS	GCCTTTGTGAACCAACACCT
	TGCTGGTTCAAGGGCTTTAT
hFABP2	TGCAGCTCATGACAATTTGA
	CCCTGAGTTCAGTTCCGTCT
hCDX2	GCAGAGCAAAGGAGAGGAAA
	AAGGGCTCTGGGACACTTCT
hALB	CCTTTGGCACAATGAAGTGGGTAACC
	CAGCAGTCAGCCATTTCACCATAGG
hAAT	ACATTTACCCAAACTGTCCATT
	GCTTCAGTCCCTTTCTCGTC
hNKX2.1	GTACCAGGACACCATGAGGAAC
	CCATGTTCTTGCTCACGTC
hSPC	GAGGTCCTGATGGAGAGC
	GCTTAGACGTAGGCACTG
mα-SMA	GCTCTGCCTCTAGCACACAA
	GCCAGGGCTACAAGTTAAGG
mFSP1	GAGGAGGCCCTGGATGTAAT
	CTTCATTGTCCCTGTTGCTG
mCol4a1	GCAACGGTACAAAGGGAGAGAG
	CTTCATTCCTGGTAACCCTGGTG

ITGA8, integrin alpha 8; CDH11, cadherin 11; αMHC, alpha-myosin heavy chain; cTNT, cardiac troponin T; HUVEC, human umbilical vein endothelial cell; INS, insulin; FABP2, fatty acid binding protein 2; ALB, albumin; AAT, alpha-1 antitrypsin; SPC, surfactant protein C; GFAP, glial fibrillary acidic protein; α-Sma, alpha-smooth muscle actin; Fsp1, Fibroblast-specific protein 1; Col4a1, alpha-1 subunit of collagen type IV.

Supplemental Method

Immunostaining and Lectin Staining

Cells were fixed with 4% paraformaldehyde (PFA, Nacalai Tesque, Kyoto, Japan)/PBS for 10 to 20 min at 4°C. For nuclear immunostaining, the fixed cells were washed twice with PBS and incubated in blocking buffer consisting of 5% normal donkey serum (Merck, Darmstadt, Germany)/PBST (PBS/0.25% Triton X-100) for 1 h at room temperature. The primary antibodies (mouse anti-HOXD11, Abcam Biochemicals; rabbit anti-SIX2, Proteintech, Rosemont, IL, USA; rat anti-GFP, Nacalai Tesque) were diluted in blocking solution at 1:200 for anti-HOXD11 and SIX2 and 1:500 for anti-GFP and incubated with samples overnight at 4°C or 4 h at room temperature. Secondary antibody (anti-Mouse IgG-Alexa 546, anti-Rabbit IgG-Alexa 546, anti-Rabbit IgG-Alexa 488; Thermo Fisher Scientific) was diluted in blocking solution at 1:1,000 and incubated with samples for 1 h at room temperature. Whole-mount immunostaining of organ culture samples was performed as described previously[17]. The cellular aggregates on filters were cut apart from a transwell insert, transferred to 24-well plates and fixed with 4% PFA/PBS for 15 min. For cell surface immunostaining, the fixed cells were washed twice with PBS and incubated in blocking buffer consisting of 5% normal donkey serum/PBST (PBS/0.1% Triton X-100) for 1 h at room temperature. To evaluate the differentiation into proximal renal tubules, staining with biotinylated *Lotus tetragonolobus* lectin (LTL, 1:200; Vector laboratories, Burlingame, CA, USA) and Alexa Fluor 546® streptavidin (1:1,000; Thermo Fisher Scientific) was carried out. For distal renal tubules, immunostaining with anti-E-cadherin (1:200; BD Biosciences) and anti-mouse IgG-Alexa 647

(1:1,000; Thermo Fisher Scientific) was performed. Stained samples were analyzed using a BZ-X700 fluorescence microscope (Keyence, Osaka, Japan). Immunostaining against α-smooth muscle action (α-SMA) on kidney sections was performed as described previously using anti-α-SMA antibody (1:300; Sigma-Aldrich)[15].

RT-PCR and Real-time Quantitative RT-PCR (qRT-PCR)

Total RNA was isolated using an RNeasy kit (Qiagen, Valencia, CA, USA) according to the manufacturer's recommendations, followed by cDNA synthesis using standard protocols. One μg of RNA was used for reverse transcription with ReverTra Ace (TOYOBO, Osaka, Japan), and PCR was carried out using the Ex-Taq PCR kit (Takara Bio, Shiga, Japan) and a thermal cycler (Veriti 96 well Thermal Cycler, Thermo Fisher Scientific) according to the manufacturer's instructions. qRT-PCR was carried out using Step One Plus Real-Time PCR System (Thermo Fisher Scientific) and SYBR Green PCR Master Mix (Takara Bio). Relative quantification was performed against a standard curve, and the expression levels were normalized to a housekeeping gene, GAPDH. The primer sets used in this study are shown in Table S4.
